# Nationwide trends in dementia subtypes and in-hospital mortality among hospitalizations with type 2 diabetes in Spain, 2021–2024: A focus on sex disparities

**DOI:** 10.1371/journal.pone.0358874

**Published:** 2026-09-24

**Authors:** Natividad Cuadrado-Corrales, Diana María Mérida, Ana Lopez-de-Andres, Jose J. Zamorano-Leon, Rodrigo Jimenez-Garcia, David Carabantes-Alarcon, Andres Bodas-Pinedo, Valentin Hernandez-Barrera, Lucia Fuentes-Arroyo

**Affiliations:** 1 Department of Public Health and Maternal & Child Health, Faculty of Medicine, Universidad Complutense de Madrid, Madrid, Spain; 2 Instituto de Investigación Sanitaria del Hospital Clínico San Carlos (IdISSC), Madrid, Spain; 3 Department of Public Health and Maternal & Child Health, Faculty of Pharmacy, Universidad Complutense de Madrid, Madrid, Spain; 4 Preventive Medicine and Public Health Teaching and Research Unit, Faculty of Health Sciences, Universidad Rey Juan Carlos, Madrid, Spain; Ehime University Graduate School of Medicine, JAPAN

## Abstract

**Background:**

The co-occurrence of type 2 diabetes mellitus (T2DM) and dementia represents a major public health challenge in Spain, where prevalence among hospitalized patients rose from 6.9% in 2011 to 10.6% in 2020. However, post-pandemic trends and sex-specific clinical outcomes remain under-characterized. We aimed to evaluate the frequency and temporal trends of dementia subtypes, clinical profiles, and predictors of in-hospital mortality (IHM) among T2DM-related hospitalizations in Spain (2021–2024).

**Methods:**

We conducted a nationwide retrospective observational study using the Spanish National Hospital Discharge Database (RAE-CMBD) and analyzed 2,786,184 T2DM-related hospital discharge episodes recorded between 2021 and 2024. The unit of analysis was the hospitalization episode rather than the unique individual; therefore, recurrent admissions for the same person could be included as separate observations. Multivariable logistic regression identified independent predictors of dementia presence and IHM.

**Results:**

All-cause dementia was coded in 180,623 hospital episodes, corresponding to 6.48% of T2DM-related hospitalizations. This proportion was higher among hospitalizations involving women than among those involving men. Regarding specific subtypes, AD was the most frequent diagnosis with 80,174 cases (2.88% prevalence), presenting an IHM of 16.3% across the study period. VaD affected 1.44% of the sample. A remarkably strong association was found between prior stroke/TIA and the presence of VaD (aOR 5.89; 95% CI 5.69–6.09). Hospitalizations involving all-cause dementia had higher in-hospital mortality (17.06% vs. 7.22%), lower ICU admission rates (1.33% vs. 7.58%), and higher frequencies of palliative-care coding (3.76% vs. 1.65%) than hospitalizations without dementia (Table 3). They also had shorter hospital stays and lower median estimated hospitalization costs (€6,129.3 vs. €8,602.94). A comprehensive breakdown of in-hospital mortality according to specific concomitant conditions and dementia status is available in S3 Table.

**Conclusions:**

Among T2DM-related hospitalizations with dementia, female sex was associated with lower adjusted odds of in-hospital mortality. Prior stroke or transient ischemic attack was strongly associated with vascular dementia coding. Lower ICU admission and higher palliative-care coding were observed among hospitalizations involving dementia; however, the clinical reasons underlying these patterns cannot be determined from administrative discharge data.

## Introduction

In recent years, the global incidence of neurodegenerative diseases, particularly dementia, has risen significantly, driven by an aging population and the increasing prevalence of metabolic disorders such as type 2 diabetes mellitus (T2DM) [1,2]. Dementia is currently a highly prevalent progressive disorder, with nearly 10 million new cases diagnosed annually worldwide [3]. Research has consistently demonstrated a robust bi-directional relationship between T2DM and cognitive decline; individuals with T2DM are 1.5 to 2.5 times more likely to develop all-cause dementia, including its primary subtypes: Alzheimer’s disease (AD) and vascular dementia (VaD) [4,5].

In Spain, dementia prevalence among 5.2 million T2DM hospitalizations rose significantly from 6.9% in 2011 to 10.6% in 2020 [6]. This decadal trend underscores the need to assess the 2021–2024 landscape to determine if these rates stabilized or continued to escalate post-pandemic [6].

Previous studies have shown that the coexistence of T2DM and dementia is associated with increased clinical complexity and higher in-hospital mortality (IHM) [7,8]. Furthermore, evidence highlighted significant sex-related disparities, where women with T2DM presented a higher prevalence of dementia, yet female sex appeared to act as a protective factor for IHM compared to men [9]. The COVID-19 pandemic further complicated this landscape, causing an unprecedented spike in mortality among these vulnerable patients during 2020 [10,11].

Sex differences play a critical role in both the incidence and hospital outcomes of these comorbid conditions [12–14]. While women with T2DM generally face a higher relative risk of developing dementia and AD than men [15], a “survival advantage” or protective effect of female sex has been consistently observed regarding IHM, even in the presence of advanced neurodegeneration [9,16,17].

The COVID-19 pandemic introduced an unprecedented systemic stressor that disproportionately affected this vulnerable population, leading to significant spikes in IHM during 2020 [6]. While previous research in Spain has characterized these trends during the 2011–2020 and 2017–2023 periods [6,17], there is a critical need to evaluate the post-acute phase of the pandemic (2021–2024). This analysis is essential to determine if mortality rates have stabilized and how the clinical profile of these patients has evolved in the current healthcare landscape.

Therefore, the objectives of this nationwide study were: (i) to assess the prevalence and temporal trends of all-cause dementia, AD, and VaD among patients hospitalized with T2DM in Spain from 2021 to 2024; (ii) to analyse sex-based disparities in clinical characteristics and hospital outcomes; and (iii) to identify independent predictors of both the presence of dementia and IHM in this population.

## Materials and methods

### Data source and study design

We conducted a nationwide retrospective observational study using the Spanish National Hospital Discharge Database (RAE-CMBD, Registro de Actividad de Atención Especializada–Conjunto Mínimo Básico de Datos). This registry, maintained by the Spanish Ministry of Health, is mandatory and captures over 95% of all hospital admissions nationwide [18,19]. The unit of analysis was the hospital discharge episode rather than the unique patient. Consequently, all estimates reported in this study refer to hospitalizations or discharge episodes. Because the anonymized RAE-CMBD does not provide a longitudinal patient identifier, multiple hospitalizations involving the same individual during the study period may have been counted as separate observations.

Our analysis focused on the post-acute phase of the pandemic and included all hospital discharge records from January 1, 2021, to December 31, 2024.

### Selection criteria and population stratification

The study population consisted of hospitalizations for patients aged ≥40 years with a diagnosis of T2DM in any diagnostic position (ICD-10 code E11.x). We excluded episodes with missing data for sex, age, or discharge status, as well as cases of type 1 diabetes. To evaluate the clinical burden, the cohort was stratified according to the presence of all-cause dementia and its primary subtypes: AD and VaD. These conditions were identified using a specific cluster of ICD-10 codes, as detailed in S1 Table.

### Clinical variables and comorbidity assessment

To identify disparities in patient profiles and hospital outcomes, we evaluated a refined core set of clinical variables, categorized into: (i) cardiometabolic comorbidities (including hypertension, dyslipidemia, obesity, heart disease, stroke/TIA, and CKD); (ii) neurological and neuropsychiatric conditions (including Parkinson’s disease, depression, and substance use); and (iii) the acute infectious context (specifically COVID-19 diagnosis). A comprehensive list of all conditions and their corresponding ICD-10 codes is provided in S1 Table. Additionally, overall comorbidity burden was assessed using the Charlson Comorbidity Index (CCI), modified by excluding T2DM and dementia from the calculation to avoid redundancy [20,21].

### Outcome measures

The primary clinical endpoint was all-cause IHM, defined as death from any cause during the hospitalization period, regardless of the primary diagnosis at admission. Secondary outcomes included the length of hospital stay (LOHS) in days, admission to the intensive care unit (ICU), and total estimated hospitalization costs.

### Statistical analysis

Annual prevalence rates were calculated for all-cause dementia, AD, and VaD among T2DM hospitalizations, with comparisons between sexes and age groups. Descriptive data were presented as means with standard deviation (SD) for continuous variables and as percentages for categorical data.

Temporal trends in categorical outcomes were evaluated using the Cochran–Armitage test for trend, with calendar year treated as an ordered variable from 2021 to 2024. Comparisons of categorical variables between groups were performed using two-sided chi-square tests, whereas continuous variables were compared using Student’s t-test or ANOVA, as appropriate. Because the large sample size could result in statistically significant p-values for small absolute differences, the magnitude of the observed changes was considered alongside statistical significance.

To determine independent predictors of both the presence of dementia and IHM, we developed multivariable logistic regression models. These models were adjusted for demographics, CCI, and the specific comorbidities in the “core set.” Results were expressed as adjusted odds ratios (aOR) with 95% confidence intervals (CI). All analyses were performed using SPSS version 29.0 or Stata version 14, with significance set at a two-sided p < 0.05.

### Ethical considerations

The data were obtained from an anonymized administrative database provided by the Spanish Ministry of Health. Under Spanish legislation [22], the use of de-identified public registries for epidemiological research does not require specific ethics committee approval or individual patient consent. Data were accessed for research purposes on September 1, 2025. The authors had no access to information that could identify individual participants during or after data collection, as all records in the RAE-CMBD are fully anonymized by the Ministry before provision.

## Results

### Overall trends in prevalence

From 2021 to 2024, the RAE-CMBD recorded 2,786,184 T2DM-related hospitalizations among individuals aged ≥40 years. Within this population, all-cause dementia was identified in 180,623 admissions, corresponding to 6.48% of all T2DM-related hospitalizations. Across the study period, the annual prevalence of all-cause dementia ranged from 8.75% to 9.45% in women and from 4.35% to 4.84% in men, with women exhibiting a consistently higher prevalence than men in all years. Regarding specific subtypes, the annual prevalence of AD ranged between 4.25% and 4.51% in women and 1.75% and 1.90% in men, while VaD prevalence was 1.73% and 1.24%, respectively. Overall, total T2DM-related hospitalizations increased from 653,517 in 2021 to 728,951 in 2024. Although the temporal trend test was statistically significant for all-cause dementia, the absolute variation over the study period was modest, ranging from 6.16% in 2021 to 6.55% in 2024. The statistical significance of this trend should therefore be interpreted in the context of the large number of hospital episodes analyzed. The annual frequency of all-cause dementia and its primary subtypes among T2DM-related hospitalizations, stratified by sex and study year, is presented in Table 1.

**Table 1:** Annual prevalence of dementia among hospitalizations with type 2 diabetes mellitus, by sex and dementia subtype. Spain (2021–2024).

Sex	Measure	2021	2022	2023	2024	Total	p-value for temporal trend
**Women**	T2DM admissions (N)	269,140	287,381	289,973	297,350	1,143,844	
	All-cause dementia, n (%)	23,561(8.75)	27,144(9.45)	26,274(9.06)	27,475(9.24)	104,454(9.13)	<0.001
	Alzheimer disease, n (%)	11,447(4.25)	12,950(4.51)	12,825(4.42)	13,118(4.41)	50,340(4.40)	0.032
	Vascular dementia, n (%)	4,647(1.73)	5,104(1.78)	4,975(1.72)	5,108(1.72)	19,834(1.73)	0.406
**Men**	T2DM admissions (N)	384,377	407,154	419,208	431,601	1,642,340	
	All-cause dementia, n (%)	16,702(4.35)	19,721(4.84)	19,496(4.65)	20,250(4.69)	76,169(4.64)	<0.001
	Alzheimer disease, n (%)	6,728(1.75)	7,752(1.90)	7,518(1.79)	7,836(1.82)	29,834(1.82)	0.435
	Vascular dementia, n (%)	4,707(1.22)	5,223(1.28)	5,063(1.21)	5,297(1.23)	20,290(1.24)	0.359
**Total**	T2DM admissions (N)	653,517	694,535	709,181	728,951	2,786,184	
	All-cause dementia, n (%)	40,263(6.16)	46,865(6.75)	45,770(6.45)	47,725(6.55)	180,623(6.48)	<0.001
	Alzheimer disease, n (%)	18,175(2.78)	20,702(2.98)	20,343(2.87)	20,954(2.87)	80,174(2.88)	0.093
	Vascular dementia, n (%)	9,354(1.43)	10,327(1.49)	10,038(1.42)	10,405(1.43)	40,124(1.44)	0.171
**Women vs Men**	**p-value (All-cause dementia)**	<0.001	<0.001	<0.001	<0.001	<0.001	
	p-value (Alzheimer dementia)	<0.001	<0.001	<0.001	<0.001	<0.001	
	p-value (Vascular dementia)	<0.001	<0.001	<0.001	<0.001	<0.001	

T2DM, type 2 diabetes mellitus. Values are presented as n (%). For each year and sex, percentages indicate the proportion of T2DM-related hospitalizations with a coded diagnosis of each dementia subtype. Women and men were compared within each year using two-sided chi-square tests. P-values for temporal trends were calculated using the two-sided Cochran–Armitage test for trend, with calendar year treated as an ordered variable. Diagnostic definitions and ICD-10 codes are provided in S1 Table.

### Demographic and clinical profiles: The frailty and comorbidity paradox

Hospitalizations involving T2DM and comorbid dementia presented a significantly different profile than those without dementia with baseline characteristics summarized in Table 2. The dementia group was characterized by an older mean age (84.06 vs. 74.46 years) and a markedly higher proportion of women (57.83% vs. 39.89%). Detailed demographic and clinical profiles of the dementia cohort, further stratified by sex, are provided in S2 Table.

**Table 2:** Characteristics of T2DM-related hospitalizations according to all-cause dementia status, Spain, 2021–2024.

Variable	Total T2DM hospitalizations	T2DM without dementia	T2DM with all-cause dementia	p-value
N	**2,786,184**	**2,605,561**	**180,623**	**–**
Age, years (mean ± SD)	75.10 (11.39)	74.46 (11.36)	84.06 (7.27)	<0.001
Age group 40–64	516,482 (18.54)	514,083 (19.73)	2,399 (1.33)	<0.001
Age group 65–74	713,342 (25.60)	697,890 (26.78)	15,452 (8.55)	<0.001
Age group 75–84	911,665 (32.72)	844,683 (32.42)	66,982 (37.08)	<0.001
Age group ≥85	644,695 (23.14)	548,905 (21.07)	95,790 (53.03)	<0.001
Female sex	1,143,844 (41.05)	1,039,390 (39.89)	104,454 (57.83)	<0.001
CCI, mean ± SD	1.29 (1.09)	1.31 (1.09)	1.14 (1.04)	<0.001
Hypertension	1,335,572 (47.94)	1,254,753 (48.16)	80,819 (44.74)	<0.001
Dyslipidemia	1,561,227 (56.03)	1,465,574 (56.25)	95,653 (52.96)	<0.001
Obesity	477,732 (17.15)	462,677 (17.76)	15,055 (8.34)	<0.001
Ischemic heart disease	569,863 (20.45)	543,748 (20.87)	26,115 (14.46)	<0.001
Heart failure	578,251 (20.75)	539,107 (20.69)	39,144 (21.67)	<0.001
Atrial fibrillation	654,161 (23.48)	606,418 (23.27)	47,743 (26.43)	<0.001
Stroke / TIA	285,297 (10.24)	254,422 (9.76)	30,875 (17.09)	<0.001
CKD	693,360 (24.89)	640,354 (24.58)	53,006 (29.35)	<0.001
COPD	306,406 (11.00)	292,197 (11.21)	14,209 (7.87)	<0.001
PD	56,461 (2.03)	44,149 (1.69)	12,312 (6.82)	<0.001
Depression	118,276 (4.25)	106,115 (4.07)	12,161 (6.73)	<0.001
Anxiety	129,637 (4.65)	120,558 (4.63)	9,079 (5.03)	<0.001
Tobacco use	821,050 (29.47)	797,320 (30.60)	23,730 (13.14)	<0.001
Alcohol use disorder / harmful use	205,388 (7.37)	198,731 (7.63)	6,657 (3.69)	<0.001
COVID-19	171,662 (6.16)	156,563 (6.01)	15,099 (8.36)	<0.001
Palliative care	49,807 (1.79)	43,017 (1.65)	6,790 (3.76)	<0.001

T2DM, type 2 diabetes mellitus; CCI, modified Charlson Comorbidity Index; CKD, chronic kidney disease; COPD, chronic obstructive pulmonary disease; HF, heart failure; AF, atrial fibrillation; TIA, transient ischemic attack. Values are presented as n (%) unless otherwise indicated. Continuous variables were compared using two-sided Student’s t-tests and categorical variables using two-sided chi-square tests. Diagnostic definitions and ICD-10-ES codes are provided in S1 Table.

Hospitalizations involving all-cause dementia had a lower mean modified CCI score than those without dementia (1.14 vs. 1.31), as shown in Table 2. Heart failure (21.67% vs. 20.69%), atrial fibrillation (26.43% vs. 23.27%), and chronic kidney disease (29.35% vs. 24.58%) were more frequent among hospitalizations involving dementia. Prior stroke/TIA was also more frequent in hospitalizations involving dementia than in those without dementia (17.09% vs. 9.76%).

### In-hospital outcomes according to dementia status

Hospitalizations involving all-cause dementia had lower ICU admission rates (1.33% vs. 7.58%; p < 0.001), higher frequencies of palliative-care coding (3.76% vs. 1.65%; p < 0.001), shorter hospital stays, and lower median estimated hospitalization costs (€6,129.3 vs. €8,602.94; p < 0.001) than hospitalizations without dementia (Table 3). A comprehensive breakdown of in-hospital mortality according to specific concomitant conditions and dementia status is available in S3 Table.

**Table 3:** Annual in-hospital outcomes among T2DM hospitalizations by dementia status and sex, Spain (2021–2024).

		2021	2022	2023	2024	TOTAL	p-value
Men IHM	**No dementia**	28,138(7.65)	27,530(7.11)	26,367(6.60)	26,649(6.48)	108,684(6.94)	<0.001
	**All-cause dementia**	3,086(18.48)	3,432(17.40)	3,236(16.60)	3,398(16.78)	13,152(17.27)	<0.001
	**Alzheimer disease**	1,174(17.45)	1,386(17.88)	1,218(16.20)	1,287(16.42)	5,065(16.98)	0.014
	**Vascular dementia**	764(16.23)	769(14.72)	698(13.79)	802(15.14)	3,033(14.95)	0.080
Women IHM	**No dementia**	19,873(8.09)	20,591(7.91)	19,422(7.37)	19,525(7.23)	79,411(7.64)	<0.001
	**All-cause dementia**	4,086(17.34)	4,712(17.36)	4,271(16.26)	4,588(16.70)	17,657(16.90)	0.004
	**Alzheimer disease**	1,860(16.25)	2,126(16.42)	1,901(14.82)	2,114(16.12)	8,001(15.89)	0.186
	**Vascular dementia**	694(14.93)	763(14.95)	716(14.39)	767(15.02)	2,940(14.82)	0.901
Both sexes IHM	**No dementia**	48,011(7.83)	48,121(7.43)	45,789(6.90)	46,174(6.78)	188,095(7.22)	<0.001
	**All-cause dementia**	7,172(17.81)	8,144(17.38)	7,507(16.40)	7,986(16.73)	30,809(17.06)	<0.001
	**Alzheimer disease**	3,034(16.69)	3,512(16.96)	3,119(15.33)	3,401(16.23)	13,066(16.30)	0.010
	**Vascular dementia**	1,458(15.59)	1,532(14.83)	1,414(14.09)	1,569(15.08)	5,973(14.89)	0.182
Men ICU admission	**No dementia**	30,848(8.39)	34,158(8.82)	34,684(8.68)	36,033(8.76)	135,723(8.67)	<0.001
	**All-cause dementia**	314(1.88)	290(1.47)	353(1.81)	336(1.66)	1,293(1.70)	0.543
	**Alzheimer disease**	129(1.92)	118(1.54)	132(1.77)	134(1.71)	513(1.73)	0.627
	**Vascular dementia**	98(2.08)	70(1.34)	67(1.32)	82(1.55)	317(1.56)	0.052
Women ICU admission	**No dementia**	14,105(5.74)	15,479(5.95)	15,839(6.01)	16,432(6.09)	61,855(5.95)	<0.001
	**All-cause dementia**	298(1.26)	289(1.06)	250(0.95)	277(1.01)	1,114(1.07)	0.003
	**Alzheimer disease**	130(1.14)	151(1.17)	140(1.09)	147(1.12)	568(1.13)	0.773
	**Vascular dementia**	82(1.76)	52(1.02)	42(0.84)	51(1.00)	227(1.14)	<0.001
Both sexes ICU admission	**No dementia**	44,953(7.33)	49,637(7.66)	50,523(7.62)	52,465(7.70)	197,578(7.58)	<0.001
	**All-cause dementia**	612(1.52)	579(1.24)	603(1.32)	613(1.28)	2,407(1.33)	0.017
	**Alzheimer disease**	259(1.43)	269(1.30)	272(1.34)	281(1.34)	1,081(1.35)	0.593
	**Vascular dementia**	180(1.92)	122(1.18)	109(1.09)	133(1.28)	544(1.36)	<0.001
Men Palliative care	**No dementia**	5,671(1.54)	6,211(1.60)	6,909(1.73)	7,047(1.71)	25,838(1.65)	<0.001
	**All-cause dementia**	572(3.42)	682(3.46)	755(3.87)	802(3.96)	2,811(3.69)	<0.001
	**Alzheimer disease**	215(3.20)	266(3.43)	276(3.67)	325(4.15)	1,082(3.63)	0.001
	**Vascular dementia**	130(2.76)	152(2.91)	146(2.88)	153(2.89)	581(2.86)	0.749
Women Palliative care	**No dementia**	3,865(1.57)	4,229(1.63)	4,445(1.69)	4,640(1.72)	17,179(1.65)	<0.001
	**All-cause dementia**	897(3.81)	1,016(3.74)	986(3.75)	1,080(3.93)	3,979(3.81)	0.444
	**Alzheimer disease**	418(3.65)	472(3.64)	475(3.70)	516(3.93)	1,881(3.74)	0.224
	**Vascular dementia**	138(2.97)	141(2.76)	147(2.95)	163(3.19)	589(2.97)	0.410
Both sexes Palliative care	**No dementia**	9,536(1.55)	10,440(1.61)	11,354(1.71)	11,687(1.72)	43,017(1.65)	<0.001
	**All-cause dementia**	1,469(3.65)	1,698(3.62)	1,741(3.80)	1,882(3.94)	6,790(3.76)	0.007
	**Alzheimer disease**	633(3.48)	738(3.56)	751(3.69)	841(4.01)	2,963(3.70)	0.004
	**Vascular dementia**	268(2.87)	293(2.84)	293(2.92)	316(3.04)	1,170(2.92)	0.419

Values are presented as n (%). For each dementia-status category and sex stratum, percentages were calculated using the corresponding number of T2DM-related hospitalizations in that category as the denominator. P-values in the final column correspond to temporal trends across 2021–2024 and were calculated using the two-sided Cochran–Armitage test for trend, with calendar year treated as an ordered variable. T2DM, type 2 diabetes mellitus; IHM, in-hospital mortality; ICU, intensive care unit.

### Multivariable analysis of factors associated with dementia and in-hospital mortality

The results of the multivariable logistic regression models identifying factors independently associated with the presence of dementia and IHM are summarized in

Table 4 and Table 5. Subtype-specific predictors for the presence of and mortality associated with AD and VaD are detailed in S4 and S5 Tables.

**Table 4:** Multivariable logistic regression for factors associated with the presence of all-cause dementia among hospitalizations with T2DM. Spain (2021–2024).

Predictor	Overall aOR (95% CI)	p-value	Women aOR (95% CI)	p-value	Men aOR (95% CI)	p-value
2021	1		1		1	–
2022	1.05(1.04-1.07)	<0.001	1.04(1.02-1.06)	<0.001	1.07(1.04-1.09)	<0.001
2023	1.04(1.03-1.06)	<0.001	1.03(1.01-1.05)	0.005	1.06(1.03-1.08)	<0.001
2024	1.07(1.05-1.09)	<0.001	1.07(1.05-1.09)	<0.001	1.07(1.05-1.1)	<0.001
Age group 40–64	1		1	<0.001	1	–
Age group 65–74	4.86(4.65-5.07)	<0.001	5.75(5.33-6.2)	<0.001	4.42(4.2-4.67)	<0.001
Age group 75–84	16.09(15.44-16.77)	<0.001	20.35(18.92-21.87)	<0.001	14.00(13.31-14.72)	<0.001
Age group ≥85	32.75(31.43-34.14)	<0.001	41.95(39.02-45.1)	<0.001	27.69(26.31-29.14)	<0.001
CCI = 0	1		1	<0.001	1	–
CCI = 1	0.64(0.63-0.65)	<0.001	0.63(0.62-0.64)	<0.001	0.65(0.64-0.67)	<0.001
CCI ≥ 2	0.42(0.41-0.43)	<0.001	0.41(0.4-0.42)	<0.001	0.44(0.43-0.45)	<0.001
Hypertension	0.79(0.78-0.8)	<0.001	0.78(0.77-0.79)	<0.001	0.81(0.8-0.83)	<0.001
Dyslipidemia	0.94(0.93-0.95)	<0.001	0.93(0.91-0.94)	<0.001	0.97(0.95-0.98)	<0.001
Obesity	0.60(0.59-0.61)	<0.001	0.60(0.59-0.61)	<0.001	0.60(0.58-0.62)	<0.001
IHD	0.84(0.82-0.85)	<0.001	0.83(0.81-0.85)	<0.001	0.84(0.83-0.86)	<0.001
Heart failure	0.93(0.91-0.94)	<0.001	0.94(0.92-0.96)	<0.001	0.90(0.88-0.92)	<0.001
Atrial fibrillation	0.84(0.83-0.85)	<0.001	0.82(0.81-0.84)	<0.001	0.87(0.85-0.88)	<0.001
Stroke / TIA	2.35(2.32-2.39)	<0.001	2.14(2.09-2.19)	<0.001	2.61(2.55-2.67)	<0.001
CKD	1.21(1.19-1.22)	<0.001	1.25(1.22-1.27)	<0.001	1.15(1.13-1.18)	<0.001
COPD	1.12(1.1-1.14)	<0.001	1.11(1.06-1.15)	<0.001	1.12(1.1-1.15)	<0.001
PD	2.91(2.84-2.97)	<0.001	2.31(2.24-2.39)	<0.001	3.57(3.46-3.67)	<0.001
Depression	1.43(1.4-1.46)	<0.001	1.30(1.27-1.34)	<0.001	1.85(1.78-1.92)	<0.001
Anxiety	1.11(1.08-1.13)	<0.001	1.05(1.02-1.08)	<0.001	1.31(1.26-1.38)	<0.001
Tobacco use	0.70(0.69-0.71)	<0.001	0.68(0.65-0.7)	<0.001	0.71(0.69-0.72)	<0.001
Alcohol use disorder	1.35(1.32-1.39)	<0.001	1.43(1.32-1.56)	<0.001	1.32(1.28-1.36)	<0.001
COVID-19	1.23(1.21-1.26)	<0.001	1.18(1.15-1.21)	<0.001	1.29(1.26-1.33)	<0.001

**Table 5:** Multivariable logistic regression analysis of factors associated with in-hospital mortality among T2DM-related hospitalizations with all-cause dementia, Spain, 2021–2024.

	Women aOR (95% CI)	p-value	Men aOR (95% CI)	p-value	Both sexes aOR (95% CI)	p-value
2021	Reference		Reference		Reference	Reference
2022	0.98(0.94-1.03)	0.441	0.91(0.86-0.96)	<0.001	0.95(0.92-0.98)	0.003
2023	0.93(0.88-0.97)	0.001	0.88(0.83-0.93)	<0.001	0.90(0.87-0.94)	<0.001
2024	0.96(0.92-1.00)	0.078	0.89(0.85-0.94)	<0.001	0.93(0.9-0.96)	<0.001
Age group: 40–64 years	Reference		Reference		Reference	Reference
Age group: 65–74 years	1.15(0.89-1.49)	0.273	1.35(1.13-1.61)	<0.001	1.29(1.12-1.49)	0.001
Age group: 75–84 years	1.37(1.07-1.76)	0.012	1.60(1.35-1.90)	<0.001	1.54(1.34-1.77)	<0.001
Age group: ≥85 years	1.95(1.52-2.49)	<0.001	2.16(1.83-2.56)	<0.001	2.14(1.86-2.46)	<0.001
CCI = 0	Reference		Reference		Reference	Reference
CCI = 1	1.13(1.07-1.18)	<0.001	1.05(0.99-1.11)	0.095	1.09(1.06-1.13)	<0.001
CCI ≥ 2	1.29(1.21-1.39)	<0.001	1.18(1.09-1.27)	<0.001	1.24(1.18-1.31)	<0.001
Hypertension	1(0.96-1.04)	0.988	0.97(0.92-1.01)	0.201	0.98(0.96-1.02)	0.328
Dyslipidemia	0.84(0.81-0.87)	<0.001	0.85(0.82-0.88)	<0.001	0.84(0.82-0.87)	<0.001
Obesity	0.81(0.76-0.86)	<0.001	0.8(0.73-0.87)	<0.001	0.81(0.77-0.85)	<0.001
Ischemic heart disease	1.13(1.07-1.19)	<0.001	0.98(0.93-1.03)	0.438	1.05(1.01-1.09)	0.012
Heart failure	1.10(1.04-1.15)	<0.001	1.20(1.13-1.26)	<0.001	1.14(1.09-1.18)	<0.001
Atrial fibrillation	1.07(1.03-1.11)	0.001	1.1(1.05-1.15)	<0.001	1.08(1.05-1.11)	<0.001
Stroke / TIA	1.15(1.09-1.21)	<0.001	0.95(0.90-1.00)	0.056	1.05(1.01-1.09)	0.009
CKD	0.95(0.9-0.99)	0.030	0.95(0.90-1.00)	0.056	0.95(0.91-0.98)	0.003
COPD	0.86(0.78-0.96)	0.005	0.83(0.78-0.88)	<0.001	0.84(0.79-0.88)	<0.001
Parkinson’s disease	1.06(0.99-1.14)	0.106	1.01(0.95-1.08)	0.761	1.04(0.99-1.09)	0.154
Depression	0.81(0.76-0.86)	<0.001	0.71(0.64-0.79)	<0.001	0.78(0.74-0.82)	<0.001
Anxiety	0.79(0.73-0.85)	<0.001	0.75(0.66-0.85)	<0.001	0.78(0.73-0.83)	<0.001
Tobacco use	0.83(0.75-0.93)	0.001	0.86(0.82-0.9)	<0.001	0.86(0.82-0.89)	<0.001
Alcohol use disorder/harmful use	0.81(0.63-1.04)	0.092	0.98(0.91-1.06)	0.604	0.96(0.89-1.04)	0.307
COVID-19	1.25(1.18-1.32)	<0.001	1.30(1.22-1.38)	<0.001	1.27(1.22-1.32)	<0.001

Multivariable logistic regression analysis of factors associated with in-hospital mortality among T2DM-related hospitalizations with all-cause dementia, stratified by sex. Adjusted odds ratios and 95% confidence intervals were obtained from models including calendar year, age group, modified Charlson Comorbidity Index, and the prespecified clinical covariates. Reference categories are indicated in the table. All tests were two-sided; p-values below 0.001 are reported as p < 0.001.

Regarding the probability of having a dementia diagnosis, age ≥ 85 years emerged as the most powerful independent predictor (aOR 32.75; 95% CI 31.43–34.14), with women in this age group showing an even higher likelihood (aOR 41.95; 95% CI 39.02–45.1) compared to men. A prior history of stroke/TIA was strongly associated with all-cause dementia (aOR 2.35; 95% CI 2.32–2.39), and this association reached its peak for VaD with a massive aOR of 5.89 (95% CI 5.69–6.09). Other significant neurological comorbidities included PD (aOR 2.91) and depression (aOR 1.43). Notably, a higher modified CCI (CCI **≥** 2) was associated with a lower probability of being diagnosed with dementia (aOR 0.42; 95% CI 0.41–0.43), suggesting a potential “comorbidity paradox” in the clinical coding of these complex patients.

In the multivariable model of IHM among T2DM-related hospitalizations with all-cause dementia, age ≥ 85 years showed the strongest positive association with IHM (aOR 2.14; 95% CI 1.86–2.46). COVID-19 was also associated with higher odds of IHM (aOR 1.27; 95% CI 1.22–1.32). Female sex was independently associated with lower odds of IHM (aOR 0.90; 95% CI 0.87–0.92). Obesity (aOR 0.81; 95% CI 0.77–0.85) and depression (aOR 0.78; 95% CI 0.74–0.82) also showed inverse associations with IHM. In contrast to the model evaluating dementia coding, a higher comorbidity burden (CCI ≥ 2) was associated with higher odds of IHM (aOR 1.24; 95% CI 1.18–1.31).

## Discussion

In this nationwide study, we characterized the trends in dementia prevalence and hospital outcomes among patients with T2DM in Spain during the 2021–2024 period. This timeframe represents a critical post-acute phase following the COVID-19 pandemic, during which healthcare systems aimed to stabilize clinical management for chronic and neurodegenerative conditions [6,17]. Our findings provide continuity to the epidemiological characterization of this vulnerable population, highlighting persistent sex-based differences in dementia frequency and in-hospital mortality, together with associations between cardiometabolic and neuropsychiatric comorbidities and hospital outcomes.

### Trends in prevalence and clinical profiles

Our findings show that dementia diagnoses remained frequent among T2DM-related hospitalizations in Spain (6.48% overall; 9.13% in women), consolidating the upward trend observed during the 2011–2020 period [6]. This high prevalence reinforces the well-documented bidirectional relationship between metabolic disorders and neurodegeneration, where T2DM patients face a 1.5 to 2.5 times higher risk of developing cognitive impairment [4,5].

Regarding dementia subtypes, AD remains the most frequent form in our sample (2.88%), though VaD (1.44%) represents a critical complication driven by cumulative glycemic toxicity and neurovascular damage [6,20,21]. The modest annual changes observed between 2021 and 2024 suggest a phase of stabilization following the disruptive impact of the COVID-19 pandemic on hospital admission patterns [6,17].

### Sex-related differences in dementia and in-hospital mortality

A central finding of this study was the marked sex difference in dementia coding. All-cause dementia was recorded in 9.13% of T2DM-related hospitalizations involving women and 4.64% of those involving men. This difference may partly reflect the older age distribution among women and sex-related differences in dementia risk described in previous studies [13,14,22,23].

Female sex was independently associated with lower odds of in-hospital mortality among T2DM-related hospitalizations with all-cause dementia (aOR 0.90; 95% CI 0.87–0.92). Although this pattern may be described as a female survival advantage, it should not be interpreted as evidence of an intrinsic biological protective effect. Residual confounding, differences in admission patterns, unmeasured disease severity, functional status, treatment eligibility, and other clinical or social factors may have contributed to the observed association.

### Clinical profiles: The vascular and neuropsychiatric burden

Our findings revealed a lower CCI in the dementia group compared to patients without cognitive impairment (1.14 vs. 1.31), despite the former being significantly older. This ‘comorbidity paradox’ likely reflects a coding bias inherent in administrative databases, where the identification of advanced dementia or acute life-threatening complications may lead to the under-reporting of secondary chronic conditions in the discharge summary.

The strong association between prior stroke/TIA and vascular dementia coding (aOR 5.89; 95% CI 5.69–6.09) highlights the relevance of cerebrovascular disease in this population [6,20]. This pattern is consistent with the contribution of cumulative neurovascular injury to cognitive decline among people with T2DM, alongside neurodegenerative mechanisms [22,23]. However, the effectiveness of any specific cerebrovascular preventive intervention cannot be inferred from this observational analysis. Furthermore, the coexistence of PD (6.82%) and the positive association with depression (aOR 1.43) indicate a substantial neuropsychiatric comorbidity burden [17]. Recent literature suggests that the multimorbidity of diabetes and depression post-stroke doubles the risk of loss of independence and progression to dementia, aligning with the high vulnerability detected in our cohort [23–25].

### Differences in care intensity according to dementia status

Hospitalizations involving all-cause dementia had lower ICU admission rates (1.33% vs. 7.58%) and higher frequencies of palliative-care coding (3.76% vs. 1.65%) than hospitalizations without dementia. They also had shorter hospital stays and lower median estimated hospitalization costs. These findings indicate differences in the intensity and orientation of hospital care. However, the RAE-CMBD does not include information on dementia severity, baseline functional status, frailty, advance directives, patient or family preferences, ICU eligibility, treatment-limitation decisions, or the clinical rationale for palliative-care referral. Therefore, the observed coding patterns should not be interpreted as direct evidence of a systematic transition to end-of-life care [26]. The observed IHM of 17.06% represents all-cause mortality occurring during hospitalizations involving dementia and should not be interpreted as mortality directly attributable to dementia. Higher mortality frequencies were observed among hospitalizations with concomitant COVID-19 or heart failure [27,28]. However, the RAE-CMBD does not include direct measures of dementia severity, frailty, baseline functional status, acute physiological severity, or the specific cause of death. Therefore, the mechanisms underlying these differences cannot be determined from the present analysis.

### Strengths and limitations

The primary strength of this study is the use of the RAE-CMBD database, ensuring nationwide representativeness that exceeds 95% of Spanish admissions [17]. The stratification by dementia subtypes and sex provides significant clinical value beyond studies that treat dementia as a single, homogeneous entity. This granular approach is essential because neurological risks in T2DM are highly heterogeneous; treating these conditions as uniform can mask distinct pathophysiological patterns and risk clusters [29,30]. Furthermore, our clinical differentiation is supported by evidence suggesting that T2DM may contribute to neurodegeneration through non-amyloidogenic pathways, such as chronic neuroinflammation and microvascular damage, rather than solely accelerating classic Alzheimer’s pathology, making the distinction between VaD and AD clinically meaningful for targeted prevention [31]. Finally, the inclusion of a sex-sensitive lens addresses a robust determinant of cognitive decline, as the synergy between metabolic status and comorbidities like depression has been shown to manifest differently across sexes [32].

However, several limitations must be acknowledged. Clinical conditions were identified from discharge coding, which may have resulted in undercoding, particularly for milder dementia [33–35]. Residual confounding is likely because the RAE-CMBD does not capture dementia severity, cognitive or functional assessments, frailty, glycemic control, diabetes duration, socioeconomic status, medication use, advance directives, patient or family preferences, ICU eligibility, treatment-limitation decisions, or the clinical rationale for palliative-care referral. The unit of analysis was the hospital discharge episode rather than the unique individual; therefore, the same person may have contributed more than one hospitalization, and within-person correlation arising from recurrent hospitalizations could not be accounted for. Consequently, the reported percentages should be interpreted as episode-level frequencies among T2DM-related hospitalizations and not as estimates of population prevalence among unique persons.

Finally, the observational and episode-based design precludes causal inference [36,37].

## Conclusions

Dementia diagnoses remained frequent among T2DM-related hospitalizations in Spain between 2021 and 2024. Women had a higher frequency of dementia coding but lower adjusted odds of in-hospital mortality than men. Prior stroke or TIA was strongly associated with vascular dementia coding. Differences in ICU admission and palliative-care coding were observed according to dementia status, although the underlying clinical reasons could not be assessed. These findings describe nationwide hospital patterns and do not establish causal relationships.

## Supporting information

S1 Table Diagnostic and procedural codes from the ICD-10 classification system employed in this study (2021–2024). (DOCX)

S2 Table Demographic and clinical characteristics and in-hospital outcomes among patients hospitalized with type 2 diabetes mellitus (T2DM) and all-cause dementia in Spain (2021–2024), according to sex. (DOCX)

S3 Table In-hospital mortality among men and women hospitalized with type 2 diabetes, according to selected concomitant condition and to the presence of all-cause dementia in Spain for the period 2021–2024. (DOCX)

S4 Table Multivariable analysis of the factors associated with the presence of AD among men and women hospitalized with T2DM and factors associated with IHM among patients with T2DM and concomitant AD. (DOCX)

S5 Table Multivariable analysis of the factors associated with the presence of VaD among men and women hospitalized with T2DM and factors associated with IHM among patients with T2DM and concomitant VaD. Spain 2021–2024.
(DOCX)
