# Supplementary material for: Nationwide trends in dementia subtypes and in-hospital mortality among hospitalizations with type 2 diabetes in Spain, 2021–2024: A focus on sex disparities
Source: PLoS One. 2026 Sep 24;21(9):e0358874. doi: 10.1371/journal.pone.0358874 (PMC13606990; doi:10.1371/journal.pone.0358874)
Supplement: Supplementary file 1 [file pone.0358874.s001.docx]

S1 Table. Diagnostic and procedural codes from the ICD-10 classification system employed in this study (2021–2024).

| CONDITION | ICD-10 CODES |
| --- | --- |
| Type 2 Diabetes Mellitus (T2DM) | E11.x |
| All-cause dementia | G30.0, G31.0, G31.83, F00, F01, F02, F03, F05.1 |
| Alzheimer dementia | G30.0, G30.1, G30.8, G30.9 |
| Vascular dementia | F01.50, F01.51 |
| Cardiometabolic Comorbidities |  |
| Hypertension | I10 |
| Dyslipidemia | E78 |
| Obesity | E66 |
| Ischemic heart disease | I20–I25 |
| Heart failure | I50 |
| Atrial fibrillation | I48 |
| Stroke / TIA | I60–I69, G45 |
| Chronic kidney disease (CKD) | N18 |
| Chronic obstructive pulmonary disease (COPD) | J44 |
| Neurological and Neuropsychiatric Comorbidities |  |
| Parkinson’s Disease | G20 |
| Depression | F32 |
| Anxiety | F40–F48 |
| Tobacco Use | F17, Z72.0, Z87.891, T65.2 |
| Alcohol use disorder / harmful use | F10 (optional: Z72.1) |
| Hypoglycemia | E10.641, E10.649 |
| Diabetic ketoacidosis | E11.x |
| COVID-19 | B34.2, B97.29, U07.1 |
| Encounter for palliative care | Z51.5 |
