# Supplementary material for: Nationwide trends in dementia subtypes and in-hospital mortality among hospitalizations with type 2 diabetes in Spain, 2021–2024: A focus on sex disparities
Source: PLoS One. 2026 Sep 24;21(9):e0358874. doi: 10.1371/journal.pone.0358874 (PMC13606990; doi:10.1371/journal.pone.0358874)
Supplement: Supplementary file 2 [file pone.0358874.s002.docx]

S2 Table. Demographic and clinical characteristics and in-hospital outcomes among patients hospitalized with type 2 diabetes mellitus (T2DM) and all-cause dementia in Spain (2021–2024), according to sex.

|  | Women | Men | All | p-value |
| --- | --- | --- | --- | --- |
| Total T2DM hospitalizations (N) | 1,143,844 | 1,642,340 | 2,786,184 | <0.001 |
| Dementia Subtypes, n (%) |  |  |  |  |
| All-cause dementia | 104454(9.13) | 76169(4.64) | 180623(6.48) | <0.001 |
| Alzheimer’s dementia | 50340(4.4) | 29834(1.82) | 80174(2.88) | <0.001 |
| Vascular dementia | 19834(1.73) | 20290(1.24) | 40124(1.44) | <0.001 |
| Demographics |  |  |  |  |
| Age, years (mean ± SD) | 85.32(6.78) | 82.25(7.43) | 84.03(7.22) | <0.001 |
| 40–64 years, n (%) | 791(0.71) | 1,681(2.09) | 2,472(1.29) | <0.001 |
| 65–74 years, n (%) | 6,530(5.86) | 9,784(12.19) | 16,314(8.51) |  |
| 75–84 years, n (%) | 37,008(33.23) | 34,708(43.25) | 71,716(37.43) |  |
| ≥85 years, n (%) | 67,030(60.19) | 34,075(42.46) | 101,105(52.77) |  |
| CCI, mean ± SD | 1.02(0.97) | 1.29(1.11) | 1.13(1.04) | <0.001 |
| Cardiometabolic Comorbidities, n (%) |  |  |  |  |
| Hypertension | 50,656(45.49) | 35,713(44.5) | 86,369(45.08) | <0.001 |
| Dyslipidemia | 58,725(52.73) | 42,575(53.05) | 101,300(52.87) | <0.001 |
| Obesity | 10,917(9.8) | 4,859(6.05) | 15,776(8.23) | <0.001 |
| Ischemic heart disease | 11,833(10.63) | 15,704(19.57) | 27,537(14.37) | <0.001 |
| Heart failure | 26,601(23.89) | 14,594(18.19) | 41,195(21.5) | <0.001 |
| Atrial fibrillation | 29,928(26.88) | 20,470(25.51) | 50,398(26.3) | <0.001 |
| Stroke / TIA | 16,355(14.69) | 15,679(19.54) | 32,034(16,72) | <0.001 |
| Chronic kidney disease (CKD) | 32,345(29.05) | 23,476(29.25) | 55,821(29,13) | 0.321 |
| COPD | 3,447(3.1) | 11,441(14.26) | 14,888(7.77) | <0.001 |
| Neurological and Neuropsychiatric, n (%) |  |  |  |  |
| Parkinson’s disease | 5,738(5.15) | 6,946(8.66) | 12,684(6.62) | <0.001 |
| Depression | 9,299(8.35) | 3,437(4.28) | 12,736(6.65) | <0.001 |
| Anxiety | 7,189(6.46) | 2,345(2.92) | 9,534(4.98) | <0.001 |
| Tobacco use | 3,498(3.14) | 21,445(26.72) | 24,943(13.02) | <0.001 |
| Alcohol use disorder / harmful use | 687(0.62) | 6,185(7.71) | 6,872(3.59) | <0.001 |
| ICU admission, n (%) | 1,227(1.11) | 1,403(1.76) | 2,630(1.38) | <0.001 |
| In-hospital mortality (IHM), n (%) | 18,734(16.82) | 13,886(17.3) | 32,620(17.02) | 0.006 |
| Length of hospital stay (days), median [IQR] | 6(6) | 6(8) | 6(7) | <0.001 |
