# Supplementary material for: Nationwide trends in dementia subtypes and in-hospital mortality among hospitalizations with type 2 diabetes in Spain, 2021–2024: A focus on sex disparities
Source: PLoS One. 2026 Sep 24;21(9):e0358874. doi: 10.1371/journal.pone.0358874 (PMC13606990; doi:10.1371/journal.pone.0358874)
Supplement: Supplementary file 3 [file pone.0358874.s003.docx]

S3 Table. In-hospital mortality among men and women hospitalized with type 2 diabetes, according to selected concomitant condition and to the presence of all-cause dementia in Spain for the period 2021–2024.

| Condition | IHMwoman (%) |  |  | IHMmen (%) |  |  | IHMtotal (%) |  |  |
| --- | --- | --- | --- | --- | --- | --- | --- | --- | --- |
|  | Without Dementia | With Dementia | p-value | Without Dementia | With Dementia | p-value | Without Dementia | With Dementia | p-value |
| Total, mean (SD) | 79,411(7.69) | 18,734(16.82) | <0.001 | 108,684(6.96) | 13,886(17.3) | <0.001 | 188,095(7.25) | 3,2620(17.02) | <0.001 |
| 40–64 y, n (%) | 4,899(3.02) | 75(9.48) | <0.001 | 12,367(3.52) | 162(9.64) | <0.001 | 17,266(3.36) | 237(9.59) | <0.001 |
| 65–74 y, n (%) | 10,507(4.67) | 735(11.26) | <0.001 | 25,682(5.44) | 1,266(12.94) | <0.001 | 36,189(5.19) | 2,001(12.27) | <0.001 |
| 75–84 y, n (%) | 24,091(7.04) | 5,091(13.76) | <0.001 | 38,146(7.67) | 5,383(15.51) | <0.001 | 62,237(7.41) | 10,474(14.6) | <0.001 |
| ≥85 y, n (%) | 39,914(13.19) | 12,833(19.15) | <0.001 | 32,489(13.48) | 7,075(20.76) | <0.001 | 72,403(13.32) | 19,908(19.69) | <0.001 |
| CCI mean (SD) | 1.6(1.04) | 1.14(1.01) | <0.001 | 1.88(1.15) | 1.35(1.12) | <0.001 | 1.76(1.11) | 1.23(1.06) | <0.001 |
| LOHS, n (%) | 6(8) | 5(8) | <0.001 | 7(11) | 5(9) | <0.001 | 6(10) | 5(8) | <0.001 |
| Hospital cost (€), m (IQR) | 7870,65(8346,28) | 5973,36(3096,79) | <0.001 | 9137,99(10699,41) | 6339,7(3761,45) | <0.001 | 8602,94(9795,29) | 6129,3(3400,45) | <0.001 |
| Hypert, n (%) | 39,914(13.19) | 12,833(19.15) | <0.001 | 32,489(13.48) | 7,075(20.76) | <0.001 | 72403(13.32) | 19908(19.69) | <0.001 |
| Dyslipide, n (%) | 30,429(6.13) | 8,014(15.82) | <0.001 | 43,119(5.72) | 5,756(16.12) | <0.001 | 73548(5.89) | 13770(15.94) | <0.001 |
| Obesity, n (%) | 39,935(6.91) | 9,111(15.51) | <0.001 | 54,139(6.14) | 6,770(15.9) | <0.001 | 94074(6.44) | 15881(15.68) | <0.001 |
| Ischemic heart disease, n (%) | 13,665(6.16) | 1,523(13.95) | <0.001 | 11,562(4.81) | 658(13.54) | <0.001 | 25,227(5.46) | 2,181(13.82) | <0.001 |
| Heartfailure, n (%) | 13,069(8.98) | 2,260(19.1) | <0.001 | 27,159(6.84) | 2,736(17.42) | <0.001 | 40,228(7.42) | 4,996(18.14) | <0.001 |
| Atrial fibr, n (%) | 30,931(12.35) | 5,275(19.83) | <0.001 | 32,670(11.4) | 3,111(21.32) | <0.001 | 63,601(11.84) | 8,386(20.36) | <0.001 |
| Stroke, n (%) | 28,073(10.91) | 5,607(18.73) | <0.001 | 33,019(9.53) | 3,982(19.45) | <0.001 | 61,092(10.12) | 9,589(19.03) | <0.001 |
| CKD, n (%) | 12,057(12.53) | 3,203(19.58) | <0.001 | 15,885(10.12) | 2,638(16.83) | <0.001 | 27,942(11.03) | 5,841(18.23) | <0.001 |
| COPD, n (%) | 28,533(11.19) | 5,852(18.09) | <0.001 | 36,963(9.66) | 4,324(18.42) | <0.001 | 65,496(10.27) | 10,176(18,23) | <0.001 |
| PKD, n (%) | 1900(10.53) | 961(16.75) | <0.001 | 2703(10.51) | 1184(17.05) | <0.001 | 4603(10.51) | 2145(16.91) | <0.001 |
| Depression, n (%) | 4653(6.64) | 1289(13.86) | <0.001 | 2288(6.46) | 437(12.71) | <0.001 | 6941(6.58) | 1726(13.55) | <0.001 |
| Anxiety | 4382(5.69) | 979(13.62) | <0.001 | 2452(5.69) | 304(12.96) | <0.001 | 6834(5.69) | 1283(13.46) | <0.001 |
| Tobacco, n (%) | 6198(4.69) | 420(12.01) | <0.001 | 39573(5.96) | 3181(14.83) | <0.001 | 45771(5.75) | 3601(14.44) | <0.001 |
| Alcohol use disorder n (%) | 968(6.49) | 77(11.21) | <0.001 | 12523(6.82) | 898(14,52) | <0.001 | 13491(6.8) | 975(14.19) | <0.001 |
| COVID-19, n (%) | 7840(12.51) | 1753(19.83) | <0.001 | 12550(13.52) | 1550(21.21) | <0.001 | 20390(13.11) | 3303(20.45) | <0.001 |
