# Supplementary material for: Nationwide trends in dementia subtypes and in-hospital mortality among hospitalizations with type 2 diabetes in Spain, 2021–2024: A focus on sex disparities
Source: PLoS One. 2026 Sep 24;21(9):e0358874. doi: 10.1371/journal.pone.0358874 (PMC13606990; doi:10.1371/journal.pone.0358874)
Supplement: Supplementary file 4 [file pone.0358874.s004.docx]

S4 Table**.** Multivariable analysis of the factors associated with the presence of AD among men and women hospitalized with T2DM and factors associated with IHM among patients with T2DM and concomitant AD.

|  | Presence of Alzheimer’s disease (aOR. 95% CI) | | | IHM among patients with T2DM and AD (aOR. 95% CI) | | |
| --- | --- | --- | --- | --- | --- | --- |
| Predictor | **Men** | **Women** | **Both Sexes** | **Men** | **Women** | **Both Sexes** |
| 2021 | **1** | **1** | **1** | **1** | **1** | **1** |
| 2022 | 1.02(1-1.04) | 1.02(0.99-1.04) | 1.03(0.99-1.06) | 1(0.95-1.06) | 1(0.94-1.07) | 1.01(0.93-1.1) |
| 2023 | 1.02(1-1.04) | 1.03(1.01-1.06) | 1.01(0.97-1.04) | 0.91(0.86-0.96) | 0.9(0.84-0.97) | 0.92(0.84-1.01) |
| 2024 | 1.04(1.02-1.06) | 1.05(1.02-1.08) | 1.03(1-1.07) | 0.98(0.93-1.03) | 1.01(0.94-1.08) | 0.94(0.86-1.03) |
| 40–64 years | 1 | 1 | 1 | 1 | 1 | 1 |
| 65–74 years | 8.92(8.12-9.79) | 10.54(9.08-12.24) | 7.82(6.94-8.83) | 1.2(0.88-1.63) | 1.13(0.68-1.9) | 1.26(0.86-1.85) |
| 75–84 years | 39.55(36.12-43.3) | 45.15(39.04-52.23) | 35.39(31.51-39.75) | 1.34(1-1.81) | 1.33(0.81-2.2) | 1.36(0.94-1.97) |
| ≥85 years | 67.27(61.43-73.66) | 75.3(65.1-87.1) | 61.7(54.9-69.33) | 1.85(1.37-2.5) | 1.87(1.13-3.08) | 1.81(1.25-2.63) |
| Female sex (vs. male) | 1.59(1.56-1.61) | N/A | — | 0.86(0.82-0.89) | N/A | — |
| CCI = 0 | 1 | 1 | 1 | 1 | 1 | 1 |
| CCI = 1 | 0.61(0.6-0.63) | 0.61(0.59-0.62) | 0.62(0.61-0.64) | 1.11(1.05-1.17) | 1.15(1.07-1.23) | 1.06(0.97-1.15) |
| CCI ≥ 2 | 0.38(0.37-0.39) | 0.38(0.36-0.39) | 0.38(0.37-0.4) | 1.25(1.16-1.36) | 1.39(1.25-1.54) | 1.09(0.97-1.23) |
| Cardiometabolic | 0.78(0.77-0.79) | 0.78(0.77-0.8) | 0.77(0.75-0.79) | 0.93(0.89-0.97) | 0.94(0.88-1) | 0.91(0.84-0.97) |
| Hypertension | 0.99(0.98-1.01) | 0.97(0.95-0.99) | 1.02(1-1.05) | 0.85(0.82-0.88) | 0.84(0.8-0.88) | 0.87(0.82-0.93) |
| Dyslipidemia | 0.5(0.49-0.51) | 0.51(0.5-0.53) | 0.47(0.44-0.49) | 0.83(0.77-0.9) | 0.81(0.74-0.89) | 0.88(0.75-1.03) |
| Obesity | 0.82(0.81-0.84) | 0.81(0.79-0.84) | 0.84(0.81-0.86) | 1.05(0.99-1.11) | 1.08(1-1.17) | 1.01(0.93-1.1) |
| Ischemic heart disease | 0.87(0.85-0.89) | 0.9(0.87-0.93) | 0.83(0.8-0.86) | 1.11(1.05-1.18) | 1.05(0.98-1.14) | 1.2(1.09-1.32) |
| Heart failure | 0.77(0.75-0.78) | 0.76(0.74-0.78) | 0.77(0.75-0.79) | 1.07(1.03-1.12) | 1.07(1.01-1.13) | 1.08(1-1.16) |
| Atrial fibrillation | 1.51(1.47-1.55) | 1.49(1.44-1.54) | 1.55(1.49-1.61) | 1.2(1.13-1.28) | 1.26(1.16-1.37) | 1.11(1-1.22) |
| Stroke / TIA | 1.12(1.1-1.14) | 1.17(1.13-1.2) | 1.06(1.02-1.09) | 0.91(0.86-0.96) | 0.88(0.82-0.95) | 0.94(0.86-1.02) |
| Chronic kidney disease | 1.08(1.04-1.11) | 1(0.94-1.06) | 1.12(1.08-1.16) | 0.86(0.79-0.93) | 0.85(0.72-1) | 0.89(0.8-0.98) |
| COPD | 0.78(0.77-0.79) | 0.78(0.77-0.8) | 0.77(0.75-0.79) | 0.93(0.89-0.97) | 0.94(0.88-1) | 0.91(0.84-0.97) |
| Parkinson’s disease | 0.89(0.86-0.93) | 0.85(0.8-0.9) | 0.95(0.89-1.01) | 1.03(0.92-1.16) | 1.05(0.9-1.22) | 1.02(0.86-1.21) |
| Depression | 1.17(1.13-1.21) | 1.1(1.06-1.14) | 1.48(1.39-1.57) | 0.8(0.74-0.88) | 0.83(0.76-0.92) | 0.71(0.59-0.86) |
| Anxiety | 0.89(0.86-0.92) | 0.85(0.81-0.88) | 1.06(0.98-1.14) | 0.82(0.74-0.9) | 0.81(0.73-0.91) | 0.82(0.66-1.02) |
| Tobacco use | 0.69(0.68-0.71) | 0.61(0.58-0.64) | 0.71(0.7-0.73) | 0.82(0.77-0.88) | 0.82(0.69-0.97) | 0.82(0.76-0.89) |
| Alcohol use disorder | 0.94(0.89-0.98) | 0.85(0.73-0.98) | 0.95(0.9-1) | 0.86(0.74-0.99) | 0.81(0.51-1.29) | 0.86(0.74-1) |
| COVID-19 | 1.29(1.26-1.33) | 1.23(1.19-1.27) | 1.39(1.33-1.44) | 1.26(1.18-1.34) | 1.27(1.16-1.38) | 1.24(1.13-1.37) |
| Woman | 1.59(1.56-1.61) |  |  | 0.86(0.82-0.89) |  |  |
