# Supplementary material for: Nationwide trends in dementia subtypes and in-hospital mortality among hospitalizations with type 2 diabetes in Spain, 2021–2024: A focus on sex disparities
Source: PLoS One. 2026 Sep 24;21(9):e0358874. doi: 10.1371/journal.pone.0358874 (PMC13606990; doi:10.1371/journal.pone.0358874)
Supplement: Supplementary file 5 [file pone.0358874.s005.docx]

S5 Table**.** Multivariable analysis of the factors associated with the presence of VaD among men and women hospitalized with T2DM and factors associated with IHM among patients with T2DM and concomitant VaD. Spain 2021–2024.

|  | **Presence of vascular dementia (aOR. 95% CI)** | | | **IHM among patients with T2DM and VaD (aOR. 95% CI)** | | |
| --- | --- | --- | --- | --- | --- | --- |
| **Predictor** | **Men** | **Women** | **Both Sexes** | **Men** | **Women** | **Both Sexes** |
| 2021 | 1 | 1 | 1 | 1 | 1 | 1 |
| 2022 | 1(0.97-1.03) | 1(0.96-1.04) | 1.01(0.97-1.05) | 0.92(0.85-1) | 0.99(0.89-1.11) | 0.86(0.77-0.96) |
| 2023 | 0.98(0.95-1.01) | 0.99(0.95-1.03) | 0.97(0.93-1.01) | 0.88(0.81-0.95) | 0.95(0.85-1.07) | 0.82(0.73-0.92) |
| 2024 | 1(0.97-1.02) | 1(0.96-1.04) | 0.99(0.95-1.03) | 0.96(0.89-1.04) | 1(0.9-1.12) | 0.93(0.83-1.03) |
| 40–64 years | 1 | 1 | 1 | 1 | 1 | 1 |
| 65–74 years | 3.68(3.4-3.97) | 3.51(3.04-4.05) | 3.75(3.42-4.11) | 1.33(1.01-1.76) | 1.29(0.76-2.19) | 1.34(0.97-1.85) |
| 75–84 years | 9.79(9.1-10.54) | 10.36(9.04-11.86) | 9.56(8.76-10.44) | 1.54(1.18-2) | 1.5(0.9-2.5) | 1.53(1.12-2.08) |
| ≥85 years | 17.31(16.08-18.63) | 18.84(16.45-21.57) | 16.26(14.88-17.78) | 2.05(1.58-2.67) | 2.05(1.23-3.4) | 2.01(1.47-2.74) |
| CCI = 0 | 1 | 1 | 1 | 1 | 1 | 1 |
| CCI = 1 | 0.78(0.75-0.8) | 0.78(0.74-0.81) | 0.77(0.74-0.81) | 1.05(0.96-1.14) | 1(0.88-1.13) | 1.09(0.96-1.24) |
| CCI ≥ 2 | 0.57(0.55-0.59) | 0.55(0.52-0.59) | 0.58(0.55-0.62) | 1.19(1.06-1.33) | 1.1(0.93-1.31) | 1.26(1.07-1.47) |
| Hypertension | 0.87(0.84-0.89) | 0.84(0.81-0.87) | 0.89(0.86-0.92) | 1.03(0.96-1.11) | 1.03(0.93-1.14) | 1.04(0.94-1.15) |
| Dyslipidemia | 1(0.98-1.02) | 1(0.97-1.03) | 0.99(0.97-1.02) | 0.86(0.81-0.9) | 0.86(0.79-0.93) | 0.86(0.79-0.93) |
| Obesity | 0.7(0.67-0.72) | 0.73(0.69-0.76) | 0.66(0.63-0.7) | 0.79(0.71-0.87) | 0.82(0.72-0.93) | 0.74(0.63-0.88) |
| Ischemic heart disease | 0.91(0.88-0.93) | 0.92(0.88-0.96) | 0.9(0.87-0.94) | 1.04(0.97-1.13) | 1.18(1.05-1.33) | 0.96(0.87-1.06) |
| Heart failure | 1.04(1.01-1.07) | 1.04(1-1.09) | 1.03(0.99-1.07) | 1.15(1.07-1.25) | 1.18(1.05-1.33) | 1.14(1.02-1.27) |
| Atrial fibrillation | 0.97(0.94-0.99) | 0.97(0.94-1) | 0.96(0.93-1) | 1.15(1.09-1.23) | 1.08(0.99-1.18) | 1.23(1.13-1.34) |
| Stroke / TIA | 5.54(5.4-5.68) | 5.2(5-5.4) | 5.89(5.69-6.09) | 1.03(0.96-1.1) | 1.12(1.01-1.24) | 0.95(0.87-1.05) |
| Chronic kidney disease | 1.35(1.31-1.39) | 1.42(1.36-1.48) | 1.29(1.24-1.34) | 0.98(0.9-1.06) | 0.99(0.88-1.1) | 0.98(0.88-1.09) |
| COPD | 1.13(1.09-1.17) | 1.13(1.04-1.22) | 1.14(1.09-1.19) | 0.81(0.73-0.9) | 0.82(0.65-1.04) | 0.79(0.7-0.9) |
| Parkinson’s disease | 1.5(1.43-1.58) | 1.27(1.18-1.38) | 1.7(1.59-1.81) | 0.99(0.86-1.13) | 0.91(0.72-1.13) | 1.05(0.88-1.25) |
| Depression | 1.51(1.45-1.57) | 1.39(1.33-1.46) | 1.79(1.67-1.92) | 0.73(0.65-0.83) | 0.73(0.63-0.85) | 0.73(0.59-0.91) |
| Anxiety | 1.32(1.26-1.38) | 1.28(1.21-1.35) | 1.41(1.3-1.53) | 0.77(0.67-0.88) | 0.77(0.65-0.91) | 0.77(0.6-0.98) |
| Tobacco use | 0.73(0.7-0.75) | 0.71(0.66-0.77) | 0.73(0.71-0.75) | 0.91(0.83-0.99) | 0.97(0.75-1.24) | 0.9(0.82-0.98) |
| Alcohol use disorder | 1.6(1.53-1.68) | 1.68(1.44-1.97) | 1.57(1.5-1.65) | 0.97(0.84-1.11) | 0.68(0.39-1.18) | 0.99(0.86-1.14) |
| COVID-19 | 1.19(1.15-1.24) | 1.13(1.07-1.19) | 1.26(1.19-1.32) | 1.31(1.18-1.44) | 1.15(1-1.34) | 1.45(1.27-1.65) |
| Woman | 0.99(0.97-1.01) |  |  | 0.91(0.86-0.97) |  |  |
