# Supplementary material for: Nationwide trends in dementia subtypes and in-hospital mortality among hospitalizations with type 2 diabetes in Spain, 2021–2024: A focus on sex disparities
Source: PLoS One. 2026 Sep 24;21(9):e0358874. doi: 10.1371/journal.pone.0358874 (PMC13606990; doi:10.1371/journal.pone.0358874)
Supplement: Supplementary file 6 [file pone.0358874.s007.docx]

**Response to the Academic Editor and Reviewer**

**Manuscript ID:** PONE-D-26-31799

**Manuscript title:** *Nationwide trends in dementia subtypes and in-hospital mortality among hospitalizations with type 2 diabetes in Spain, 2021–2024: a focus on sex disparities*

Dear Academic Editor and Reviewer,

We sincerely thank the Academic Editor and the Reviewer for the careful evaluation of our manuscript and for the constructive and detailed comments. We appreciate the positive assessment of the nationwide scope and overall methodological approach of the study.

We have carefully considered every comment and revised the manuscript accordingly. The revisions have improved the precision of the epidemiological terminology, the transparency and reproducibility of the statistical methods, the consistency of the tables, the description of data availability, and the interpretation of the observational findings.

All changes are marked in the tracked-changes version of the revised manuscript. Page and line references below correspond to the revised clean manuscript and should be updated after the final document has been typeset.

**Point-by-point response**

**Comment 1. Clarify the unit of analysis and epidemiological terminology**

**Reviewer comment:**
The Methods appropriately state that the unit of analysis is the hospital discharge episode rather than the individual patient. Nevertheless, terms such as “patients,” “individuals,” and “prevalence” are occasionally used in a manner that could be interpreted as referring to unique persons or population prevalence.

Please ensure that the title, abstract, tables, Results and Conclusions consistently describe the estimates as the proportion or prevalence of dementia diagnoses among T2DM-related hospitalizations. Where appropriate, “patients” should be replaced by “hospitalizations,” “admissions,” or “hospital episodes.” The possibility that the same patient contributed more than one admission should also be reiterated briefly in the limitations.

**Response:**

We thank the Reviewer for identifying this important issue. We agree that the distinction between hospital episodes and unique individuals must be maintained consistently. We have reviewed the title, Abstract, Methods, Results, table titles, Discussion, and Conclusions and replaced potentially ambiguous references to “patients” or “individuals” with “hospitalizations,” “admissions,” or “hospital discharge episodes,” where appropriate.

We have also clarified that the reported percentages represent the proportion of T2DM-related hospitalizations in which dementia was coded and do not represent population prevalence among unique persons. In addition, the possibility of recurrent hospitalizations from the same person has been explicitly included in the limitations.

**Changes introduced in the manuscript:**

**Abstract, Methods section, revised page 2, lines 25–30:**

“We conducted a nationwide retrospective observational study using the Spanish National Hospital Discharge Database (RAE-CMBD) and analyzed 2,786,184 T2DM-related hospital discharge episodes recorded between 2021 and 2024. The unit of analysis was the hospitalization episode rather than the unique individual; therefore, recurrent admissions for the same person could be included as separate observations.”

**Abstract, Results section, revised page 2, lines 32–34:**

“All-cause dementia was coded in 180,623 hospital episodes, corresponding to 6.48% of T2DM-related hospitalizations. This proportion was higher among hospitalizations involving women than among those involving men.”

**Methods, Data Source and Study Design, revised page 5, lines 98–102:**

“The unit of analysis was the hospital discharge episode rather than the unique patient. Consequently, all estimates reported in this study refer to hospitalizations or discharge episodes. Because the anonymized RAE-CMBD does not provide a longitudinal patient identifier, multiple hospitalizations involving the same individual during the study period may have been counted as separate observations.”

**Limitations, revised page 23, lines 330–342:**

“However, several limitations must be acknowledged. Clinical conditions were identified from discharge coding, which may have resulted in undercoding, particularly for milder dementia (33–35). Residual confounding is likely because the RAE-CMBD does not capture dementia severity, cognitive or functional assessments, frailty, glycemic control, diabetes duration, socioeconomic status, medication use, advance directives, patient or family preferences, ICU eligibility, treatment-limitation decisions, or the clinical rationale for palliative-care referral. The unit of analysis was the hospital discharge episode rather than the unique individual; therefore, the same person may have contributed more than one hospitalization, and within-person correlation arising from recurrent hospitalizations could not be accounted for. Consequently, the reported percentages should be interpreted as episode-level frequencies among T2DM-related hospitalizations and not as estimates of population prevalence among unique persons. Finally, the observational and episode-based design precludes causal inference (36,37).

In addition, the titles of the relevant tables and the corresponding passages in the Results and Discussion sections were revised to consistently refer to hospitalizations or hospital episodes rather than unique patients.

**Comment 2. Describe the statistical test used for temporal trends**

**Reviewer comment:**
The manuscript reports multiple “p-values for time trend,” but the Statistical Analysis section does not specify how these trends were tested. Please state whether year was entered as a continuous variable in a regression model, whether a chi-square test for linear trend was used, or whether another procedure was applied.

Given the very large sample size, the authors should also distinguish between statistical significance and the relatively modest absolute changes observed from 2021 to 2024.

**Response:**

We agree with the Reviewer that the statistical procedure used to evaluate temporal trends should have been specified more explicitly. We have revised the Statistical Analysis section to state that temporal trends in proportions were assessed using the Cochran–Armitage test for trend, with calendar year treated as an ordered variable.

We have also revised the Results and Discussion to emphasize that, despite statistically significant trend tests for several outcomes, the absolute year-to-year changes were modest. This distinction is particularly important given the very large sample size.

**Changes introduced in the manuscript:**

**Methods, Statistical Analysis, revised pages 6–7, lines 132–138:**

“Temporal trends in categorical outcomes were evaluated using the Cochran–Armitage test for trend, with calendar year treated as an ordered variable from 2021 to 2024. Comparisons of categorical variables between groups were performed using two-sided chi-square tests, whereas continuous variables were compared using Student’s t-test or ANOVA, as appropriate. Because the large sample size could result in statistically significant p-values for small absolute differences, the magnitude of the observed changes was considered alongside statistical significance.”

**Results, Overall trends in prevalence, revised page 8, lines 163–167:**

“Overall, total T2DM-related hospitalizations increased from 653,517 in 2021 to 728,951 in 2024. Although the temporal trend test was statistically significant for all-cause dementia, the absolute variation over the study period was modest, ranging from 6.16% in 2021 to 6.55% in 2024. The statistical significance of this trend should therefore be interpreted in the context of the large number of hospital episodes analyzed.”

The heading of the final column in Table 1 was revised from ‘P for time trend’ to ‘p-value for temporal trend.’ In Table 3, the existing heading ‘p-value’ was retained, and the table legend was revised to clarify that the values in the final column correspond to temporal trends across 2021–2024.

**Table 1 legend, revised page 9, lines 169–173:**

The legends of Tables 1 and 3 were revised to identify the Cochran–Armitage test:

“T2DM, type 2 diabetes mellitus. Values are presented as n (%). For each year and sex, percentages indicate the proportion of T2DM-related hospitalizations with a coded diagnosis of each dementia subtype. Women and men were compared within each year using two-sided chi-square tests. P-values for temporal trends were calculated using the two-sided Cochran–Armitage test for trend, with calendar year treated as an ordered variable. Diagnostic definitions and ICD-10 codes are provided in S1 Table.”

**Table 3 legend, revised page 13, lines 206–210:**

“This is the Table 3 legend.

Values are presented as n (%). For each dementia-status category and sex stratum, percentages were calculated using the corresponding number of T2DM-related hospitalizations in that category as the denominator. P-values in the final column correspond to temporal trends across 2021–2024 and were calculated using the two-sided Cochran–Armitage test for trend, with calendar year treated as an ordered variable. T2DM, type 2 diabetes mellitus; IHM, in-hospital mortality; ICU, intensive care unit.”

**Comment 3. Verify the values and p-values reported in Table 5**

**Reviewer comment:**
Several entries in the male-specific p-value column of Table 5 appear to be erroneous or displaced. For example, values such as 1.131, 1.354, 1.825, 1.130, 1.050 and 1.220 cannot be valid p-values and do not correspond to the reported confidence intervals.

Please carefully verify the original statistical output and correct the entire table. Values currently reported as “0.000” should be presented as “p < 0.001.” The authors should also confirm that all confidence intervals, reference categories and p-values are aligned with the correct predictors.

**Response:**
We thank the Reviewer for identifying this formatting and transcription error. After checking the output of the rerun multivariable logistic regression models, we confirmed that the values originally displayed in the male-specific p-value column had been incorrectly transferred during the preparation of Table 5. Several entries corresponded to the lower limits of the 95% confidence intervals rather than to the actual p-values.

We reconstructed Table 5 directly from the verified statistical output and independently checked all adjusted odds ratios, 95% confidence intervals, reference categories, and p-values for the overall and sex-stratified models. We also replaced all values previously reported as “0.000” with “p < 0.001” and standardized the decimal and statistical formatting throughout the table.

**Changes introduced in the manuscript, Table 5, revised pages 17–18 (caption lines 229–230; legend lines 231–235):**

Table 5 has been replaced with a corrected version. The male-specific p-values have been realigned with their corresponding predictors, invalid values greater than 1 have been removed, reference categories are now explicitly identified, and all p-values below 0.001 are reported as p < 0.001.

The revised legend reads:

“This is the Table 5 legend.

Multivariable logistic regression analysis of factors associated with in-hospital mortality among T2DM-related hospitalizations with all-cause dementia, stratified by sex. Adjusted odds ratios and 95% confidence intervals were obtained from models including calendar year, age group, modified Charlson Comorbidity Index, and the prespecified clinical covariates. Reference categories are indicated in the table. All tests were two-sided; p-values below 0.001 are reported as p < 0.001.”

In addition, the paragraph immediately following Table 5 was revised to ensure consistency with the corrected model estimates and to avoid causal terminology. Specifically, expressions such as “risk factor,” “increased the risk,” and “protective factor” were replaced with association-based language. The estimate for female sex reported in the narrative Results was also verified against the corrected overall model and updated accordingly. This estimate is distinct from the coefficient for tobacco use in the overall model, which was verified as aOR 0.86 (95% CI 0.82–0.89; p < 0.001).

**Changes introduced in the manuscript, Table 5 and accompanying Results paragraph, revised pages 17–19 (Table 5: lines 229–235; accompanying Results: lines 236–243):**

“In the multivariable model of IHM among T2DM-related hospitalizations with all-cause dementia, age ≥85 years showed the strongest positive association with IHM (aOR 2.14; 95% CI 1.86–2.46). COVID-19 was also associated with higher odds of IHM (aOR 1.27; 95% CI 1.22–1.32). Female sex was independently associated with lower odds of IHM (aOR 0.90; 95% CI 0.87–0.92). Obesity (aOR 0.81; 95% CI 0.77–0.85) and depression (aOR 0.78; 95% CI 0.74–0.82) also showed inverse associations with IHM. In contrast to the model evaluating dementia coding, a higher comorbidity burden (CCI ≥2) was associated with higher odds of IHM (aOR 1.24; 95% CI 1.18–1.31).”

**Comment 4. Reconcile denominators and proofread all tables**

**Reviewer comment:**
The denominators in Table 2 require verification. The reported numbers for hospitalizations without dementia and with all-cause dementia do not sum to the total number of T2DM hospitalizations:

2,594,577 + 180,623 = 2,775,200, rather than 2,786,184.

Please explain whether the difference represents hospitalizations with another dementia classification, missing dementia status, overlapping diagnostic categories, or a transcription error.

A comprehensive proofreading of Tables 1–5 is also required. Examples include misplaced digits in admission totals, “admisions,” “Alzeimer,” “Paliative care,” “Bot sexes,” inconsistent decimal precision and inconsistent spacing around percentages. All totals and percentages should be recalculated and cross-checked against the narrative Results.

**Response:**

We thank the Reviewer for carefully identifying the inconsistency in the denominators reported in Table 2. After re-examining the analytic dataset, we confirmed that the number originally reported for T2DM-related hospitalizations without all-cause dementia resulted from a transcription error during table preparation. The discrepancy did not represent missing dementia status, an additional dementia classification, or overlapping study groups.

The correct study-group sizes are 2,786,184 total T2DM-related hospitalizations, comprising 2,605,561 hospitalizations without all-cause dementia and 180,623 hospitalizations with all-cause dementia. We reran the descriptive analyses using these mutually exclusive groups and regenerated Table 2 directly from the final analytic dataset. All counts, percentages, means, standard deviations, and p-values were recalculated and cross-checked. For every categorical variable, the counts in the two dementia-status groups now reconcile with the corresponding overall total. The cross-check also led to minor corrections of percentages in Table 3 and the corresponding values reported in the Abstract, Results, and Discussion, ensuring complete numerical consistency across the manuscript.

We also revised the title of Table 2 to reflect that the unit of analysis was the hospitalization episode rather than the unique individual. The p-value was removed from the row reporting the group sample sizes because no statistical comparison is applicable to that row.

In addition, we conducted a comprehensive proofreading of Tables 1–5. Misplaced digits in admission totals and typographical errors, including “admisions,” “Alzeimer,” “Paliative care,” and “Bot sexes,” were corrected. Terminology, abbreviations, decimal precision, spacing between counts and percentages, reference categories, and statistical formatting were standardized throughout the tables. All table totals and percentages were cross-checked against the corresponding values reported in the Abstract, Results, and Discussion.

**Changes introduced in the manuscript:**

**Table 2, revised page 10, lines 182–183:**

“Table 2. Characteristics of T2DM-related hospitalizations according to all-cause dementia status, Spain, 2021–2024.”

The corrected study-group sizes are now reported as follows:

“Total T2DM-related hospitalizations: 2,786,184; hospitalizations without all-cause dementia: 2,605,561; hospitalizations with all-cause dementia: 180,623.”

All row-level counts, percentages, means, standard deviations, and p-values in Table 2 were recalculated using the corrected mutually exclusive groups and verified against the corresponding overall totals.

**Table 2 legend, revised pages 10–11, lines 184–190:**

The Table 2 legend was also revised to standardize terminology, abbreviations, statistical-test descriptions, and the reference to the ICD-10-ES codes. The revised legend reads:

“T2DM, type 2 diabetes mellitus; CCI, modified Charlson Comorbidity Index; CKD, chronic kidney disease; COPD, chronic obstructive pulmonary disease; HF, heart failure; AF, atrial fibrillation; TIA, transient ischemic attack. Values are presented as n (%) unless otherwise indicated. Continuous variables were compared using two-sided Student’s t-tests and categorical variables using two-sided chi-square tests. Diagnostic definitions and ICD-10-ES codes are provided in S1 Table.”

**Tables 1–5, revised pages 8–9, 10–11, 12–13, 15, and 17–18:**

“Typographical errors, misplaced digits, inconsistent decimal precision, and spacing errors were corrected throughout the tables. Terminology and abbreviations were standardized, and all reported totals and percentages were cross-checked against the narrative Results.”

**Comment 5. Strengthen the Data Availability Statement**

**Reviewer comment:**
The statement that RAE-CMBD data can be obtained through a formal request to the Spanish Ministry of Health is appropriate, but it should be made more operational. Please provide the specific webpage or application route, the name or version of the requested dataset, and any relevant eligibility or data-use conditions.

**Response:**

We agree with the Reviewer and have expanded the Data Availability Statement to provide a more operational description of the data-access procedure. A new standalone section entitled “Data Availability Statement” has been added immediately before the References section of the revised manuscript. The corresponding statement in the submission system has also been updated to ensure consistency.

The revised statement identifies the dataset as the Registro de Actividad de Atención Especializada–Conjunto Mínimo Básico de Datos (RAE-CMBD), specifies the study period of 2021–2024, identifies the Spanish Ministry of Health as the responsible institution, and provides direct links to the official application webpage and application form.

The record-level hospital discharge microdata cannot be redistributed by the authors under the conditions governing their provision. Researchers may apply directly to the Spanish Ministry of Health. Applicants must specify the requested period, variables, record-selection criteria, research objectives, proposed analyses, and dissemination plan, and must accept the applicable confidentiality and data-use conditions. The authors had no special access privileges that would not be available to other researchers submitting an equivalent request.

The ICD-10-ES codes used to define T2DM, dementia subtypes, comorbidities, and hospital outcomes are provided in S1 Table.

**Changes introduced in the manuscript:**

A new standalone section entitled “Data Availability Statement” was added immediately after the Funding section and before the References section.

**Data Availability Statement, revised pages 24–25, lines 366–385:**

“The record-level hospital discharge data analyzed in this study were obtained from the Registro de Actividad de Atención Especializada–Conjunto Mínimo Básico de Datos (RAE-CMBD), Spanish National Hospital Discharge Database, for the years 2021–2024. The authors are not authorized to redistribute this microdata under the conditions governing their provision by the Spanish Ministry of Health. Researchers may request access directly from the Spanish Ministry of Health through the official RAE-CMBD data application procedure available at: <https://www.sanidad.gob.es/estadEstudios/estadisticas/estadisticas/estMinisterio/SolicitudCMBD.htm>. The official application form is available at: <https://www.sanidad.gob.es/estadEstudios/estadisticas/estadisticas/estMinisterio/SolicitudCMBDdocs/Formulario_Peticion_Datos_RAE_CMBD.pdf>.

Applicants must specify the requested study period, variables, record-selection criteria, research objectives, proposed analyses, and dissemination plan, and must accept the applicable confidentiality and data-use conditions. The authors had no special access privileges that would not be available to other researchers submitting an equivalent request. The ICD-10-ES codes used to define T2DM, dementia subtypes, comorbidities, and hospital outcomes are provided in S1 Table.

**Comment 6. Moderate causal and interpretative statements**

**Reviewer comment:**
Some statements go beyond what can be concluded from an observational analysis of administrative discharge records. Expressions such as “the most effective strategy,” “biological resilience,” “clinical pivot,” “terminal stage,” and “aggressive secondary prevention” should be revised or qualified.

The lower frequency of ICU admission and higher frequency of palliative-care coding are important observations, but the database does not contain information on dementia severity, advance directives, patient preferences, clinical eligibility for intensive care or decision-making processes. The authors should therefore use formulations such as “patterns consistent with a shift toward less intensive care” rather than implying that the reasons for these decisions have been established.

Similarly, “female survival advantage” may be retained as descriptive terminology, but it should be made clear that residual confounding, differences in admission selection and unmeasured clinical severity may contribute to the association.

**Response:**

We thank the Reviewer for this important observation. We agree that several statements in the original manuscript went beyond what can be supported by an observational analysis of administrative hospital discharge records.

We therefore conducted a comprehensive revision of the Abstract, Results, Discussion, Limitations, and Conclusions. Expressions implying causality, biological protection, treatment effectiveness, terminal disease recognition, or established clinical decision-making were removed. Specifically, the terms ‘biological resilience,’ ‘independent protective factor,’ ‘most effective strategy,’ ‘aggressive secondary prevention,’ ‘clinical pivot,’ ‘terminal stage,’ and ‘adequacy of therapeutic effort’ were removed or replaced with neutral, association-based language. The term ‘female survival advantage’ was retained only as descriptive terminology and was explicitly qualified to acknowledge residual confounding, differences in admission patterns, unmeasured disease severity, functional status, treatment eligibility, and other clinical or social factors.

We also revised statements characterizing dementia as a proxy for “extreme frailty,” “advanced systemic decline,” or “severely diminished physiological reserve,” because the RAE-CMBD does not contain direct measures of dementia severity, frailty, baseline functional status, acute physiological severity, advance directives, treatment eligibility, patient or family preferences, or the clinical decision-making processes governing ICU admission and palliative-care referral.

The revised manuscript now distinguishes clearly between the observed hospital coding patterns and the clinical mechanisms or decisions that might underlie them. We also expanded the Limitations section to acknowledge residual confounding, the absence of relevant clinical variables, the episode-based unit of analysis, and the inability to draw causal conclusions.

**Changes introduced in the manuscript:**

**a. Abstract, Results**

**Revised page 2, lines 38–44:**

The statements describing female sex as a protective factor and the observed care patterns as a “clinical pivot” were removed.

They were replaced with:

“Hospitalizations involving all-cause dementia had higher in-hospital mortality (17.06% vs. 7.22%), lower ICU admission rates (1.33% vs. 7.58%), and higher frequencies of palliative-care coding (3.76% vs. 1.65%) than hospitalizations without dementia (Table 3). They also had shorter hospital stays and lower median estimated hospitalization costs (€6,129.3 vs. €8,602.94). A comprehensive breakdown of in-hospital mortality according to specific concomitant conditions and dementia status is available in S3 Table.”

**b. Abstract, Conclusions, revised page 3, lines 45–51:**

The original interpretative conclusion was replaced with:

“Among T2DM-related hospitalizations with dementia, female sex was associated with lower adjusted odds of in-hospital mortality. Prior stroke or transient ischemic attack was strongly associated with vascular dementia coding. Lower ICU admission and higher palliative-care coding were observed among hospitalizations involving dementia; however, the clinical reasons underlying these patterns cannot be determined from administrative discharge data.”

**c. Results, in-hospital outcomes, revised page 11, lines 198–204:**

The original paragraph describing a “clinical pivot toward comfort-oriented care” was removed.

It was replaced with:

“Hospitalizations involving all-cause dementia had higher in-hospital mortality (17.06% vs. 7.22%), lower ICU admission rates (1.33% vs. 7.58%), and higher frequencies of palliative-care coding (3.76% vs. 1.65%) than hospitalizations without dementia (Table 3). They also had shorter hospital stays and lower median estimated hospitalization costs (€6,129.3 vs. €8,602.94).”

**d. Discussion, Sex-related differences, revised page 20, lines 273–279:**

The following sentence was removed:

“This finding remains significant even after adjusting for the fact that women in our cohort were significantly older and had a higher prevalence of dementia subtypes, suggesting a biological or clinical resilience that persists despite the double burden of metabolic and cognitive decline.”

**It was replaced with:**

“Female sex was independently associated with lower odds of in-hospital mortality among T2DM-related hospitalizations with all-cause dementia (aOR 0.90; 95% CI 0.87–0.92). Although this pattern may be described as a female survival advantage, it should not be interpreted as evidence of an intrinsic biological protective effect. Residual confounding, differences in admission patterns, unmeasured disease severity, functional status, treatment eligibility, and other clinical or social factors may have contributed to the observed association.”

**e. Discussion, vascular dementia, revised page 21, lines 287–293:**

**The original paragraph:**

“The massive association identified between prior stroke/TIA and the presence of VaD (aOR 5.89; 95% CI 5.69–6.09) underscores a critical window for intervention. Unlike AD, where the association with vascular events was significantly lower (aOR 1.55), our data suggest that in the T2DM population, aggressive secondary prevention of cerebrovascular damage remains the most effective strategy to mitigate the rising incidence of vascular-type cognitive decline (6,20). These findings highlight that dementia in the diabetic patient is often a cumulative result of neurovascular injury rather than a purely neurodegenerative process (22,23).”

**was replaced with:**

“The strong association between prior stroke/TIA and vascular dementia coding (aOR 5.89; 95% CI 5.69–6.09) highlights the relevance of cerebrovascular disease in this population (6,20). This pattern is consistent with the contribution of cumulative neurovascular injury to cognitive decline among people with T2DM, alongside neurodegenerative mechanisms (22,23). However, the effectiveness of any specific cerebrovascular preventive intervention cannot be inferred from this observational analysis.”

**f. Discussion, care intensity, revised pages 21–22, lines 298–307:**

**The original subsection heading:**

“Shifting Care Intensity: Palliative Care vs. ICU Admission”

**was replaced with:**

“Differences in Care Intensity According to Dementia Status”

**The original paragraph:**

“Our results reveal a significant divergence in the intensity of inpatient care based on cognitive status. Patients with dementia were nearly six times less likely to be admitted to the ICU (1.34% vs. 7.66) but were more than twice as likely to receive palliative care (coded as Z51.5) (3.76% vs. 1.66%) compared to those without dementia. This lower utilization of intensive resources, combined with shorter hospital stays (5 vs. 6 days) and significantly lower median hospitalization costs (€6,129.3 vs. €8,602.94), does not indicate lower clinical severity. Instead, it reflects the advanced and terminal nature of many admissions in this group, where clinical recognition of dementia as a terminal stage likely guides the transition toward comfort-oriented care (26).

A critical observation in our study is the marked divergence between the high IHM (17.06%) and the relatively low absolute rate of palliative care coding (3.76%) among patients with dementia. While an absolute rate of 3.76% might initially suggest under-treatment at the end of life, the relative increase is telling: these patients were more than twice as likely to receive palliative interventions than those without dementia. When viewed alongside the six-fold lower rate of ICU admissions, a clear ‘clinical pivot’ emerges. These patterns suggest that the Spanish healthcare system is increasingly recognizing advanced dementia in T2DM patients as a terminal stage, prioritizing comfort-oriented pathways over aggressive, invasive interventions.”

**was replaced with:**

“Hospitalizations involving all-cause dementia had lower ICU admission rates (1.33% vs. 7.58%) and higher frequencies of palliative-care coding (3.76% vs. 1.65%) than hospitalizations without dementia. They also had shorter hospital stays and lower median estimated hospitalization costs. These findings indicate differences in the intensity and orientation of hospital care. However, the RAE-CMBD does not include information on dementia severity, baseline functional status, frailty, advance directives, patient or family preferences, ICU eligibility, treatment-limitation decisions, or the clinical rationale for palliative-care referral. Therefore, the observed coding patterns should not be interpreted as direct evidence of a systematic transition to end-of-life care (26).”

**g. Discussion, interpretation of in-hospital mortality, revised page 22, lines 307–314:**

**The paragraph beginning:**

“It is fundamental to interpret the identified 17.06% IHM…”

and ending:

“…infections or cardiovascular events become life-threatening triggers.”

**was replaced with:**

“The observed IHM of 17.06% represents all-cause mortality occurring during hospitalizations involving dementia and should not be interpreted as mortality directly attributable to dementia. Higher mortality frequencies were observed among hospitalizations with concomitant COVID-19 or heart failure. However, the RAE-CMBD does not include direct measures of dementia severity, frailty, baseline functional status, acute physiological severity, or the specific cause of death. Therefore, the mechanisms underlying these differences cannot be determined from the present analysis.”

**h. Limitations, revised page 23, lines 330–342:**

**The original limitations paragraph**:

“However, several limitations must be noted. As an administrative database study, identifying conditions relies on discharge reports, which may lead to the undercoding of dementia, particularly in its milder stages (33–35). Additionally, the lack of clinical variables such as the duration of T2DM, exact HbA1c levels, or outpatient pharmacological treatments precludes a deeper analysis of glycemic control's influence on cognitive outcomes (20). Finally, the cross-sectional nature of the data limits the ability to draw definitive causal inferences regarding the timing of disease progression (36,37).”

**was replaced with:**

“However, several limitations must be acknowledged. Clinical conditions were identified from discharge coding, which may have resulted in undercoding, particularly for milder dementia (33–35). Residual confounding is likely because the RAE-CMBD does not capture dementia severity, cognitive or functional assessments, frailty, glycemic control, diabetes duration, socioeconomic status, medication use, advance directives, patient or family preferences, ICU eligibility, treatment-limitation decisions, or the clinical rationale for palliative-care referral. The unit of analysis was the hospital discharge episode rather than the unique individual; therefore, the same person may have contributed more than one hospitalization, and within-person correlation arising from recurrent hospitalizations could not be accounted for. Finally, the observational and episode-based design precludes causal inference (36,37).”

**i. Conclusions, revised page 23, lines 343–350:**

The Conclusions section was replaced in its entirety. The expressions “female survival advantage,” “independent protective factor,” “critical clinical window,” “aggressive secondary prevention,” “clinical pivot,” “terminal stage,” and “adequacy of therapeutic effort” were removed.

**The revised Conclusions read:**

“Dementia diagnoses remained frequent among T2DM-related hospitalizations in Spain between 2021 and 2024. Women had a higher frequency of dementia coding but lower adjusted odds of in-hospital mortality than men. Prior stroke or TIA was strongly associated with vascular dementia coding. Differences in ICU admission and palliative-care coding were observed according to dementia status, although the underlying clinical reasons could not be assessed. These findings describe nationwide hospital patterns and do not establish causal relationships.”

**Closing statement**

We are grateful to the Academic Editor and the Reviewer for their thoughtful and constructive assessment. We believe that the revisions have substantially improved the methodological transparency, internal consistency, reproducibility, and interpretative precision of the manuscript.

We hope that the revised version satisfactorily addresses all the concerns raised, and we respectfully submit it for further consideration for publication in PLOS ONE.

Sincerely,

**Jose J. Zamorano-León**
Corresponding Author
On behalf of all authors
