# Supplementary material for: Nationwide trends in dementia subtypes and in-hospital mortality among hospitalizations with type 2 diabetes in Spain, 2021–2024: A focus on sex disparities
Source: PLoS One. 2026 Sep 24;21(9):e0358874. doi: 10.1371/journal.pone.0358874 (PMC13606990; doi:10.1371/journal.pone.0358874)
Supplement: Peer review history

# Peer review: Decision Letter 0

**DOI:** 10.1371/journal.pone.0358874.r001

**Authors:** Masaki Mogi

5 Aug 2026

Dear Dr. Zamorano-Leon,

Thank you for submitting your manuscript to PLOS ONE. After careful consideration, we feel that it has merit but does not fully meet PLOS ONE’s publication criteria as it currently stands. Therefore, we invite you to submit a revised version of the manuscript that addresses the points raised during the review process.

==============================

==============================

Please submit your revised manuscript by Oct 04 2026 11:59PM. If you will need more time than this to complete your revisions, please reply to this message or contact the journal office at plosone@plos.org. When you're ready to submit your revision, log on to https://www.editorialmanager.com/pone/ and select the 'Submissions Needing Revision' folder to locate your manuscript file.

- A letter that responds to each point raised by the academic editor and reviewer(s). You should upload this letter as a separate file labeled 'Response to Reviewers'.
- A marked-up copy of your manuscript that highlights changes made to the original version. You should upload this as a separate file labeled 'Revised Manuscript with Track Changes'.
- An unmarked version of your revised paper without tracked changes. You should upload this as a separate file labeled 'Manuscript'.

If you would like to make changes to your financial disclosure, please include your updated statement in your cover letter. Guidelines for resubmitting your figure files are available below the reviewer comments at the end of this letter.

If applicable, we recommend that you deposit your laboratory protocols in protocols.io to enhance the reproducibility of your results. Protocols.io assigns your protocol its own identifier (DOI) so that it can be cited independently in the future. For instructions see: https://journals.plos.org/plosone/s/submission-guidelines#loc-laboratory-protocols. Additionally, PLOS ONE offers an option for publishing peer-reviewed Lab Protocol articles, which describe protocols hosted on protocols.io. Read more information on sharing protocols at https://plos.org/protocols?utm_medium=editorial-email&utm_source=authorletters&utm_campaign=protocols.

As the corresponding author, your ORCID iD is verified in the submission system and will appear in the published article. PLOS supports the use of ORCID, and we encourage all coauthors to register for an ORCID iD and use it as well. Please encourage your coauthors to verify their ORCID iD within the submission system before final acceptance, as unverified ORCID iDs will not appear in the published article. *Only*  the individual author can complete the verification step; PLOS staff *cannot*  verify ORCID iDs on behalf of authors.

We look forward to receiving your revised manuscript.

Kind regards,

Masaki Mogi

Academic Editor

PLOS One

**Journal Requirements:**

When submitting your revision, we need you to address these additional requirements.

1. Please ensure that your manuscript meets PLOS ONE's style requirements, including those for file naming. The PLOS ONE style templates can be found at

https://journals.plos.org/plosone/s/file?id=wjVg/PLOSOne_formatting_sample_main_body.pdf and

https://journals.plos.org/plosone/s/file?id=ba62/PLOSOne_formatting_sample_title_authors_affiliations.pdf

2. Thank you for stating the following financial disclosure:

“This work has been supported by the by Universidad Complutense de Madrid, Grupo de Investigación en Epidemiología de las Enfermedades Crónicas de Alta Prevalencia en España (970970) and by Universidad Complutense de Madrid, Grupo de Investigación en Biomedicina Predictiva e Investigación Traslacional en las Enfermedades Respiratorias, Cardiovasculares y Metabólicas (970793).”

Please state what role the funders took in the study.  If the funders had no role, please state: "The funders had no role in study design, data collection and analysis, decision to publish, or preparation of the manuscript."

If this statement is not correct you must amend it as needed.

Please include this amended Role of Funder statement in your cover letter; we will change the online submission form on your behalf.

3. Please note that funding information should not appear in any section or other areas of your manuscript. We will only publish funding information present in the Funding Statement section of the online submission form. Please remove any funding-related text from the manuscript.

4. In the online submission form, you indicated that your data is available only on request from a third party. Please note that your Data Availability Statement is currently missing contact details for the third party, such as an email address or a link to where data requests can be made. Please update your statement with the missing information.

5. Please include captions for your Supporting Information files at the end of your manuscript, and update any in-text citations to match accordingly. Please see our Supporting Information guidelines for more information: http://journals.plos.org/plosone/s/supporting-information.

6. If the reviewer comments include a recommendation to cite specific previously published works, please review and evaluate these publications to determine whether they are relevant and should be cited. There is no requirement to cite these works unless the editor has indicated otherwise.

[Note: HTML markup is below. Please do not edit.]

Reviewers' comments:

Reviewer's Responses to Questions

**Comments to the Author**

1. Is the manuscript technically sound, and do the data support the conclusions?

Reviewer #1: Yes

**********

2. Has the statistical analysis been performed appropriately and rigorously?

Reviewer #1: Yes

**********

3. Have the authors made all data underlying the findings in their manuscript fully available?

The PLOS Data policy

Reviewer #1: Yes

**********

4. Is the manuscript presented in an intelligible fashion and written in standard English?

Reviewer #1: Yes

**********

Reviewer #1: This nationwide retrospective observational study examines 2,786,184 hospital discharge episodes involving patients with type 2 diabetes mellitus in Spain between 2021 and 2024. The authors assess episode-level prevalence of all-cause dementia, Alzheimer’s disease and vascular dementia, evaluate sex-related differences in hospital outcomes, and identify factors associated with dementia coding and in-hospital mortality.

The study is based on a large national database, addresses a clearly defined research question, and applies generally appropriate statistical methods. The principal results are supported by the analyses presented. However, several methodological clarifications, numerical corrections and revisions of interpretative language are required to improve transparency, reproducibility and consistency.

MINOR COMMENTS

1. Clarify the unit of analysis and epidemiological terminology: the Methods appropriately state that the unit of analysis is the hospital discharge episode rather than the individual patient. Nevertheless, terms such as “patients,” “individuals,” and “prevalence” are occasionally used in a manner that could be interpreted as referring to unique persons or population prevalence.

Please ensure that the title, abstract, tables, Results and Conclusions consistently describe the estimates as the proportion or prevalence of dementia diagnoses among T2DM-related hospitalizations. Where appropriate, “patients” should be replaced by “hospitalizations,” “admissions,” or “hospital episodes.” The possibility that the same patient contributed more than one admission should also be reiterated briefly in the limitations.

2. Describe the statistical test used for temporal trends: the manuscript reports multiple “p-values for time trend,” but the Statistical Analysis section does not specify how these trends were tested. Please state whether year was entered as a continuous variable in a regression model, whether a chi-square test for linear trend was used, or whether another procedure was applied.

Given the very large sample size, the authors should also distinguish between statistical significance and the relatively modest absolute changes observed from 2021 to 2024.

3. Verify the values and p-values reported in Table 5: several entries in the male-specific p-value column of Table 5 appear to be erroneous or displaced. For example, values such as 1.131, 1.354, 1.825, 1.130, 1.050 and 1.220 cannot be valid p-values and do not correspond to the reported confidence intervals.

Please carefully verify the original statistical output and correct the entire table. Values currently reported as “0.000” should be presented as “p < 0.001.” The authors should also confirm that all confidence intervals, reference categories and p-values are aligned with the correct predictors.

4. Reconcile denominators and proofread all tables: the denominators in Table 2 require verification. The reported numbers for hospitalizations without dementia and with all-cause dementia do not sum to the total number of T2DM hospitalizations:

2,594,577 + 180,623 = 2,775,200, rather than 2,786,184.

Please explain whether the difference represents hospitalizations with another dementia classification, missing dementia status, overlapping diagnostic categories, or a transcription error.

A comprehensive proofreading of Tables 1–5 is also required. Examples include misplaced digits in admission totals, “admisions,” “Alzeimer,” “Paliative care,” “Bot sexes,” inconsistent decimal precision and inconsistent spacing around percentages. All totals and percentages should be recalculated and cross-checked against the narrative Results.

5. Strengthen the Data Availability Statement: the statement that RAE-CMBD data can be obtained through a formal request to the Spanish Ministry of Health is appropriate, but it should be made more operational. Please provide the specific webpage or application route, the name or version of the requested dataset, and any relevant eligibility or data-use conditions.

6. Moderate causal and interpretative statements: some statements go beyond what can be concluded from an observational analysis of administrative discharge records. In particular, expressions such as “the most effective strategy,” “biological resilience,” “clinical pivot,” “terminal stage,” and “aggressive secondary prevention” should be revised or qualified.

The lower frequency of ICU admission and higher frequency of palliative-care coding are important observations, but the database does not contain information on dementia severity, advance directives, patient preferences, clinical eligibility for intensive care or decision-making processes. The authors should therefore use formulations such as “patterns consistent with a shift toward less intensive care” rather than implying that the reasons for these decisions have been established.

Similarly, “female survival advantage” may be retained as descriptive terminology, but it should be made clear that residual confounding, differences in admission selection and unmeasured clinical severity may contribute to the association.

**********

what does this mean?). If published, this will include your full peer review and any attached files.

If you choose “no”, your identity will remain anonymous but your review may still be made public.

**Do you want your identity to be public for this peer review?**  For information about this choice, including consent withdrawal, please see our Privacy Policy

Reviewer #1: No

**********

[NOTE: If reviewer comments were submitted as an attachment file, they will be attached to this email and accessible via the submission site. Please log into your account, locate the manuscript record, and check for the action link "View Attachments". If this link does not appear, there are no attachment files.]

To ensure your figures meet our technical requirements, please review our figure guidelines: https://journals.plos.org/plosone/s/figures

You may also use PLOS’s free figure tool, NAAS, to help you prepare publication quality figures: https://journals.plos.org/plosone/s/figures#loc-tools-for-figure-preparation.

NAAS will assess whether your figures meet our technical requirements by comparing each figure against our figure specifications.

# Author comment: Author response to Decision Letter 1

**DOI:** 10.1371/journal.pone.0358874.r002

3 Sep 2026

We thank the Academic Editor and Reviewer for their careful and constructive comments. We have addressed all points raised and revised the manuscript accordingly. A detailed point-by-point response, including the corresponding page and line numbers in the revised manuscript, has been uploaded as “Response to Reviewers.”

Attachment Submitted filename: Response to Reviewers.docx

# Peer review: Decision Letter 1

**DOI:** 10.1371/journal.pone.0358874.r003

**Authors:** Masaki Mogi

7 Sep 2026

Nationwide trends in dementia subtypes and in-hospital mortality among hospitalizations with type 2 diabetes in Spain, 2021–2024: a focus on sex disparities

PONE-D-26-31799R1

Dear Dr. Zamorano-Leon,

We’re pleased to inform you that your manuscript has been judged scientifically suitable for publication and will be formally accepted for publication once it meets all outstanding technical requirements.

Within one week, you’ll receive an e-mail detailing the required amendments. When these have been addressed, you’ll receive a formal acceptance letter and your manuscript will be scheduled for publication.

An invoice will be generated when your article is formally accepted. Please note, if your institution has a publishing partnership with PLOS and your article meets the relevant criteria, all or part of your publication costs will be covered. Please make sure your user information is up-to-date by logging into Editorial Manager at Editorial Manager® and clicking the ‘Update My Information' link at the top of the page. For questions related to billing, please contact billing support.

If your institution or institutions have a press office, please notify them about your upcoming paper to help maximize its impact. If they’ll be preparing press materials, please inform our press team as soon as possible -- no later than 48 hours after receiving the formal acceptance. Your manuscript will remain under strict press embargo until 2 pm Eastern Time on the date of publication. For more information, please contact onepress@plos.org.

Kind regards,

Masaki Mogi

Academic Editor

PLOS One

Additional Editor Comments (optional):

Reviewers' comments:

Reviewer's Responses to Questions

**Comments to the Author**

Reviewer #1: All comments have been addressed

**********

2. Is the manuscript technically sound, and do the data support the conclusions?

Reviewer #1: Yes

**********

3. Has the statistical analysis been performed appropriately and rigorously?

Reviewer #1: Yes

**********

4. Have the authors made all data underlying the findings in their manuscript fully available?

The PLOS Data policy

Reviewer #1: Yes

**********

5. Is the manuscript presented in an intelligible fashion and written in standard English?

Reviewer #1: Yes

**********

Reviewer #1: The authors have adequately addressed all the issues raised. Accordingly, the manuscript is suitable for acceptance in its current form.

**********

what does this mean?). If published, this will include your full peer review and any attached files.

If you choose “no”, your identity will remain anonymous but your review may still be made public.

**Do you want your identity to be public for this peer review?**  For information about this choice, including consent withdrawal, please see our Privacy Policy

Reviewer #1: No

**********

# Editor report: Acceptance letter

**DOI:** 10.1371/journal.pone.0358874.r004

**Authors:** Masaki Mogi

PONE-D-26-31799R1

PLOS One

Dear Dr. Zamorano-Leon,

I'm pleased to inform you that your manuscript has been deemed suitable for publication in PLOS One. Congratulations! Your manuscript is now being handed over to our production team.

At this stage, our production department will prepare your paper for publication. This includes ensuring the following:

* All references, tables, and figures are properly cited

* All relevant supporting information is included in the manuscript submission,

* There are no issues that prevent the paper from being properly typeset

You will receive further instructions from the production team, including instructions on how to review your proof when it is ready. Please keep in mind that we are working through a large volume of accepted articles, so please give us a few days to review your paper and let you know the next and final steps.

Lastly, if your institution or institutions have a press office, please let them know about your upcoming paper now to help maximize its impact. If they'll be preparing press materials, please inform our press team within the next 48 hours. Your manuscript will remain under strict press embargo until 2 pm Eastern Time on the date of publication. For more information, please contact onepress@plos.org.

You will receive an invoice from PLOS for your publication fee after your manuscript has reached the completed accept phase. If you receive an email requesting payment before acceptance or for any other service, this may be a phishing scheme. Learn how to identify phishing emails and protect your accounts at https://explore.plos.org/phishing.

If we can help with anything else, please email us at customercare@plos.org.

Thank you for submitting your work to PLOS One and supporting open access.

Kind regards,

PLOS One Editorial Office Staff

on behalf of

Dr. Masaki Mogi

Academic Editor

PLOS One
